# Air Pollution and Temperature in Seizures and Epilepsy: A Scoping Review of Epidemiological Studies

**DOI:** 10.1007/s40572-024-00466-3

**Published:** 2024-12-10

**Authors:** Rachit Sharma, Leah H. Schinasi, Brian K. Lee, Jennifer Weuve, Marc G. Weisskopf, Perry E. Sheffield, Jane E. Clougherty

**Affiliations:** 1 Dornsife School of Public Health, Drexel University, Philadelphia, PA 19104 USA; 2 Urban Health Collaborative, Drexel University, Philadelphia, PA 19104 USA; 3 Boston University School of Public Health, Boston University, Boston, MA 02118 USA; 4 Harvard T.H. Chan School of Public Health, Harvard University, Boston, MA 02115 USA; 5 Icahn School of Medicine at Mount Sinai, New York, NY 10029 USA

**Keywords:** Air pollution, Temperature, Climate change, Seizures, Epilepsy, Review

## Abstract

**Purpose of the Review:**

Seizures and epilepsy can be debilitating neurological conditions and have few known causes. Emerging evidence has highlighted the potential contribution of environmental exposures to the etiology of these conditions, possibly manifesting via neuroinflammation and increased oxidative stress in the brain. We conducted a scoping review of epidemiological literature linking air pollution and temperature exposures with incidence and acute aggravation of seizures and epilepsy. We systematically searched PubMed, Embase, Web of Science, and APA PsycINFO databases for peer-reviewed journal articles published in English from inception to February 7, 2024.

**Recent Findings:**

We identified a total of 34 studies: 16 examined air pollution exposure, 12 ambient temperature, and six examined both air pollution and ambient temperature. Most studies were conducted in Asia (China, Taiwan, South Korea, and Japan). Nearly all studies retrospectively derived acute (daily average), ambient, and postnatal exposure estimates from ground monitoring systems and ascertained epilepsy cases or seizure events through record linkage with medical records, health registry systems, or insurance claims data. Commonly assessed exposures were particulate matter (PM_2.5_, PM_10_), nitrogen dioxide (NO_2_), sulfur dioxide (SO_2_), carbon monoxide (CO), ozone (O_3_), and daily mean ambient temperature. Overall, the main findings across studies lacked consistency, with mixed results reported for the associations of air pollutants and temperature metrics with both seizure incidence and acute aggravations of epilepsy.

**Supplementary Information:**

The online version contains supplementary material available at 10.1007/s40572-024-00466-3.

## Abstract (summary)

An increasing number of studies have explored the relation of air pollution and temperature exposure with seizures and epilepsy. Research addressing the limitations of existing evidence is needed to arrive at more definitive causal conclusions.

**Supplementary Information:**

The online version contains supplementary material available at 10.1007/s40572-024-00466-3.

## Introduction

Seizures and epilepsy can be debilitating neurological conditions imposing a significant burden on global health. A seizure is “a transient occurrence of signs and/or symptoms due to abnormal excessive or synchronous neuronal activity in the brain” [1] that can manifest in various ways, affecting behavior, consciousness, or motor function. Epilepsy, on the other hand, is a chronic condition characterized by at least two unprovoked seizures separated by at least 24 h [1]. It was estimated that in 2016, 45.9 million people worldwide had active epilepsy, with prevalence varying by age, peaking among children aged 5–9 years and individuals aged over 80 years [2]. Seizures and epilepsy can have significant physical and psychological consequences, including injuries and accidents from seizures to comorbidities such as depression, anxiety, cognitive impairment, and social stigma [3, 4]. These conditions also carry a substantial economic burden, including direct medical costs from emergency department visits, hospitalizations, diagnostic tests, and medications, as well as indirect costs such as lost productivity, unemployment, and decreased quality of life for both persons living with the condition and their caregivers [5]. In 2019, the total epilepsy costs around the world were estimated to be $119.27 billion (2019 USD value), with the average cost per person with epilepsy estimated at $4,467 per year, ranging from $204 in low-income countries to $11,432 in high-income countries [5].

The International League Against Epilepsy (ILAE) provides separate classification systems for seizures [6] and epilepsy [7]. Seizures are largely classified based on clinical and electroencephalographic (EEG) features into focal onset seizures (subtypes: simple or complex), generalized onset seizures (subtypes: absence, tonic, atonic, clonic, myoclonic, or tonic-clonic), unknown onset seizures, and unclassified seizures [6]. Epilepsies – of which the majority are idiopathic [2] – are classified based on a combination of clinical features, seizure types, and possible etiology into focal epilepsies (subtypes: with known or unknown cause), generalized epilepsies (subtypes: genetic, developmental and epileptic encephalopathy, or with febrile seizures plus), combined focal and generalized epilepsies, unknown epilepsies, and epilepsy types by etiology (infectious etiology e.g., cerebral malaria, cerebral toxoplasmosis, and congenital infections such as Zika virus and cytomegalovirus, structural etiology through stroke or head/brain trauma, or genetic, metabolic, immune, or unknown etiologies) [7]. Determining the underlying cause of epilepsy and its progression is crucial for effective prevention, management, and treatment of the condition [8, 9].

Exposure to air pollution from anthropogenic sources and extreme ambient temperatures (both hot and cold) are among the primary environmental contributors of health disparities worldwide, leading to worsening health conditions under a rapidly changing global climate [10, 11]. Epidemiological and experimental studies have linked exposure to air pollution and extreme temperatures to a range of neurological and psychiatric disorders, such as Parkinson’s disease, dementia, autism spectrum disorder, suicidal behavior, depression, and anxiety [12–15]. These exposures have also been implicated in their potential role in causing as well as clinically aggravating or triggering seizures and epilepsy [16, 17]. Air pollution and high-temperature exposure may induce neuroinflammation and increase oxidative stress in the brain, the primary pathophysiology underlying seizures and epilepsy risk [18, 19]. Brain neuroinflammation and oxidative stress are integral to several mechanistic pathways that lead to seizures and epilepsy including astrocytic and microglial activation, release of cytokines (IL-1β, IL-6, and TNF-α) and pro-inflammatory enzymes (COX2), reactive oxygen and nitrogen species liberation, and increased NMDA receptor expression in the brain [16, 17]. Moreover, perturbed pathways related to systemic inflammation and oxidative stress, nucleic acid damage and repair, and metabolic and immune functions have been identified with both short-term and long-term exposures to air pollution [20–22] and ambient temperatures [23, 24].

A growing number of epidemiological studies have quantified the relationship of environmental exposure to air pollution and temperature with seizures or epilepsy outcomes. Although some narrative reviews have drawn attention to such studies [16, 17], few if any reviews have systematically identified and summarized their main characteristics, including the epidemiological study designs and analytical approaches adopted, and key findings. To provide a more comprehensive understanding of this literature, we conducted a scoping review. For this review, we carried out a structured search of multiple databases to identify and summarize epidemiological studies examining the relationship of air pollution and temperature exposures with incidence as well as acute aggravation of seizures and epilepsy outcomes. We also highlight gaps in the existing literature and provide guidance for future research.

## Methods

We followed the Preferred Reporting Items for Systematic Reviews and Meta-Analyses extension for scoping reviews (PRISMA-ScR) [25] to review the literature examining the relationships between environmental exposure to air pollution and temperature and seizures and epilepsy outcomes. We searched four electronic databases—MEDLINE (Ovid), Web of Science, Embase, and APA PsycINFO—for peer-reviewed studies published in English from the inception of the databases to February 7, 2024. Relevant search terms and concepts were identified to capture the key elements of the research topic and were combined using appropriate Boolean operators (AND, OR) and truncation symbols to expand or narrow the search scope as needed. The search terms used in each database are listed in Supplementary Table 1.

Two authors (R.S. and J.E.C.) evaluated the eligibility of titles and abstracts using Rayyan [26], a web-based screening tool, following the removal of duplicate publications. Any questions or discrepancies were resolved by consensus with the other authors. After the initial screening, a thorough review of the full text of each potentially eligible article was independently conducted by R.S. and J.E.C. No restrictions by air pollution type, temperature metric, seizure and epilepsy type, geography, or demography were applied. However, studies were excluded if they were not peer-reviewed, empirical, or human population-based (reviews, commentaries/editorials, conference abstracts, dissertations, case studies, and non-human studies), or published in languages other than English. Data were extracted from each included study on the study location and duration, study design and analytical approach used, characteristics of the study population and outcome(s), exposure measures along with spatiotemporal resolution and assessment method(s), covariate/ adjustment variables, and the main findings quantifying the associations between exposures and outcomes.

## Results

**Fig. 1:**
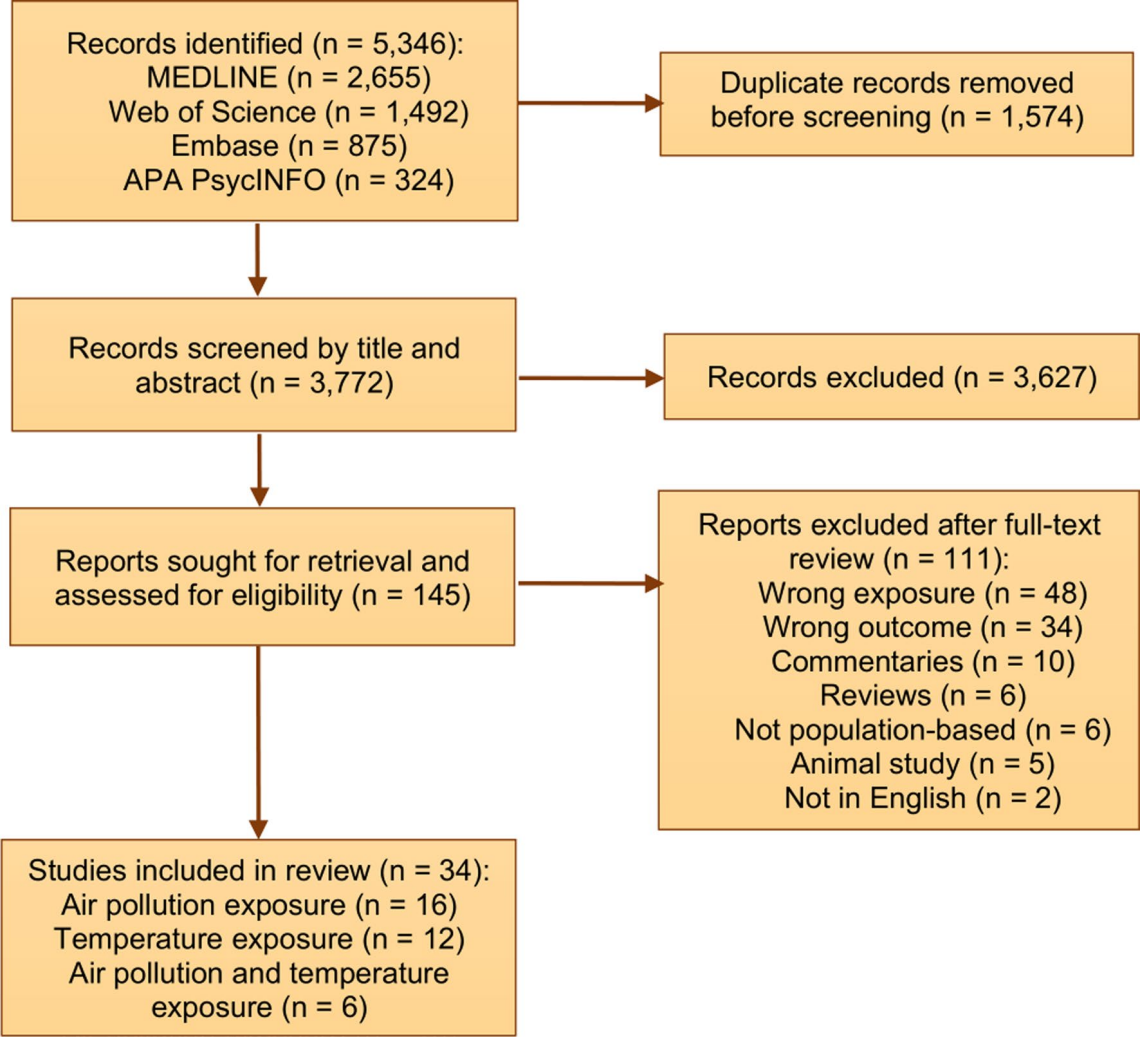
Flowchart of literature search

We found 5,346 articles across MEDLINE (2,655), Web of Science (1,492), Embase (875), and APA PsycINFO (324) as of February 7, 2024 (Fig. 1). After removing duplicates (*n* = 1,574), we screened 3,772 articles by title and abstract for eligibility. This resulted in 145 articles, of which full texts were reviewed. After applying the exclusion criteria and conducting manual searches within the references, we were left with 34 articles that were deemed relevant for final inclusion in the review. As the main outcome of interest, of the 34 articles, 18 focused on seizures [27–44] and 15 focused on epilepsy [45–59], and one focused on both seizures and epilepsy [60]. Among exposures, 16 of the total 34 articles examined relationships with air pollution [28, 30, 36, 39, 45, 47, 49–55, 57, 59, 60], 12 with ambient temperature [27, 31, 33, 35, 37, 38, 40, 41, 44, 46, 48, 56], and 6 with both air pollution and ambient temperature [29, 32, 34, 42, 43, 58].

While the earliest study was published in 2008, most of the studies (*n* = 29) appeared from 2017 onwards. The majority of the studies were conducted across Asia [China (*n* = 7), Taiwan (*n* = 3), South Korea (*n* = 5), Japan (*n* = 2)], followed by parts of Europe [UK (*n* = 2), Germany (*n* = 2), Switzerland (*n* = 1), Denmark (*n* = 1), Portugal (*n* = 1)], North America [USA (*n* = 3), Canada (*n* = 1)], the Middle East [Iran (*n* = 2), Turkey(*n* = 1)], South America [Chile (*n* = 1), Brazil (*n* = 1)], and Australia (*n* = 1).

Details on the individual study location and duration, study design and analytical approach, study population and outcome(s), exposure(s), exposure resolution and assessment method, covariate(s), and main findings can be found in Supplementary Tables S2 and S3.

### Study Populations and Outcomes

Of the 18 studies on seizures, seven focused exclusively on epileptic seizures [27–29, 35, 37, 40, 44], followed by four on febrile seizures [30, 32, 34, 41], two on convulsions [36, 43], two on generally defined unprovoked seizures [39, 42], one on psychogenic nonepileptic seizures (PNES) [31], one on status epilepticus [38], and one on febrile seizures, afebrile seizures, epileptic seizures, and status epilepticus [33]. Febrile seizures are typically associated with fever and triggered by a rapid rise in body temperature, often due to an infection. Afebrile seizures occur without a fever and are often linked with epilepsy, head injuries, genetic factors, or other neurological conditions. The term “convulsion” is a commonly used but ambiguous and unofficial term that refers to significant motor activity during a seizure. PNES include sudden changes in motor activity, sensation, behavior, or autonomic signs but unlike epileptic seizures, PNES do not involve abnormal electrical discharges in the brain or exhibit electrographical changes in the EEG. Status epilepticus involves either a single prolonged seizure lasting more than 5 min or recurrent seizures without the person regaining consciousness between episodes. Studies on epilepsy often included all forms of epilepsy, and while most focused on seizures and epilepsy outcomes among adult population groups, 10 studies focused exclusively on children [30, 32–34, 41–43, 49, 53, 58], and 10 included all ages [29, 35, 36, 44, 45, 47, 51, 56, 57, 59]. Three studies exclusively focused on the first diagnoses of febrile seizures in children [30, 32, 41], and one performed a subgroup analysis among adults with first diagnoses of unprovoked seizures and those with previous/repeat seizures [27].

Most of the studies (*n* = 31) identified individuals with seizures or epilepsy or emergency department (ED)/ hospital visits for these conditions through record linkage with medical records, nationwide (or, in some studies, statewide) healthcare registry systems or health insurance claims databases. These databases were considered representative of the general population largely due to universal health coverage in these study settings. The studies used the International Classification of Diseases, Ninth (ICD-9) and/or Tenth (ICD-10) revision medical codes, or their country-specific equivalents to identify cases/ED visits for seizures and epilepsy. Among the other studies, one study [60] observed the number of medical emergency calls for epilepsy at a medical emergency center. Only one study [28], conducted in Australia, involved active case ascertainment for epileptic seizures, wherein the authors recorded 6,692 epileptic seizure events (in a panel of 49 known epileptic patients) using long-term intracranial electroencephalography (iEEG) and a seizure diary mobile application. Additionally, only one study [46] focused on mortality associated with epilepsy, where the authors identified cases of sudden unexpected death in epilepsy (SUDEP) in England and Wales from their national clinical audit of epilepsy-related deaths.

The sample sizes for seizure-related outcomes ranged from 49 individuals with epilepsy accounting for 6,692 epileptic seizure events in Australia to 180,175 individuals with epilepsy accounting for 1,010,027 epileptic seizure events across Taiwan. The sample sizes for epilepsy ranged from 545 individuals diagnosed with epilepsy at two hospitals in Iran to 290,500 individuals with epilepsy who presented at hospitals across seven urban centers in Chile.

### Exposures

Several studies have examined the effects of multiple air pollutants, including nitrogen dioxide (NO_2_, *n* = 16), particulate matter with an aerodynamic diameter of 10 μm or less (PM_10_, *n* = 15), sulfur dioxide (SO_2_, *n* = 14), fine particulate matter with an aerodynamic diameter of 2.5 μm or less (PM_2.5_, *n* = 12), ozone (O_3_, *n* = 12), carbon monoxide (CO, *n* = 11), coarse particulate matter with an aerodynamic diameter between 2.5 μm and 10 μm (PM_10 − 2.5,_*n* = 2), nitrous oxide (N_2_O, *n* = 1), methane (CH_4_, *n* = 2), nitric oxide (NO, *n* = 2), photochemical oxidant (OX, *n* = 1), and nonmethane hydrocarbons (NMHC, *n* = 1). One study [53] also assessed exposure to heavy metals such as lead (Pb), cadmium (Cd), chromium (Cr), copper (Cu), manganese (Mn), iron (Fe), nickel (Ni), and arsenic (As). Some studies used the Air Quality Index (AQI) [43], proximity to industrial zones [50], and severe haze event [60] as measures of air pollution exposure. Among the ambient temperature exposure metrics, most studies used daily mean temperature measurements (Tmean, *n* = 18), followed by daily minimum temperature (Tmin, *n* = 6) and daily maximum temperature (Tmax, *n* = 5).

Nearly all studies examined the effects of acute (daily) air pollution and/or ambient temperature exposures that were assessed during the post-natal period. Only two studies [30, 53] examined effects of exposure during the pre-natal period; one study [53] assigned prenatal exposures to multiple air pollutants and heavy metals averaged across each trimester of pregnancy, while another [30] assigned both prenatal and postnatal (up to 6 years after birth) exposures to traffic-related air pollution. Four studies [30, 36, 53, 55], including the two pre-natal studies, examined the effects of longer-term (annual or monthly average) air pollution exposures, which were ascertained using ground-monitoring data [53], dispersion modeling [30], Land Use Regression (LUR) modeling [55], and a machine learning model that used satellite data, meteorological variables, land-use variables, elevation, chemical transport model predictions, and several reanalysis datasets [36]. The remaining majority of the studies derived spatiotemporal exposure estimates (mostly as city-level, daily averages) from air pollution ground monitoring systems and weather/ meteorological stations. All studies retrospectively ascertained ambient exposures often assigned at the residence level using residential addresses at the time of encounter with the healthcare system/recruitment.

### Study Designs, Analytical Approaches, and Covariates

Of the total 34 studies, 21 studies [27, 29, 31–35, 38, 40–43, 47, 48, 52, 54, 56–60] used the time-series design generally involving the analytical approaches of Poisson or quasi-Poisson generalized linear or additive regression models. Of these 21 time-series studies, one was annual [52], one was monthly [48], one was weekly [32], and the remaining 18 were daily time-series analyses. Six studies [28, 37, 39, 44, 45, 49] utilized the case-crossover design generally applying conditional logistic or conditional Poisson regressions. One of the six case-crossover studies was bidirectional [37] and the remaining were time-stratified. Nearly all time-series and case-crossover studies fit distributed lag models (DLMs) to describe exposure-response relationships across lagged periods of time (delayed effects). Three time-series studies [33, 34, 56] and one case-crossover study [44] fit distributed lag non-linear models (DLNMs) to simultaneously describe non-linear and lagged effects. Another study [35] used a combination of time-series data mining methods (ensemble empirical mode decomposition and Fourier–Gaussian decomposition) also suited for describing such non-linear relationships. To adjust for potential time-variant covariates like relative humidity, which could also have non-linear effects, the case-crossover and time-series studies often fit them as natural cubic or penalized spline functions. Four studies were retrospective cohorts, two of which conducted time-to-event analyses using the Cox proportional-hazards regression models [30, 50], one applied unconditional logistic regression [53], and one compared observed vs. expected number of epilepsy-related deaths using chi-square goodness of fit test [46]. Two studies were ecological, one of which fit Poisson generalized additive model [51] and another fit an unconstrained distributed lag linear model and a Bayesian hierarchical model with a Poisson distribution [36]. One study was cross-sectional [55] and applied unconditional logistic regression.

Among studies that adjusted for individual-level covariates, four studies [30, 50, 53, 55] that were either retrospective cohort or cross-sectional by design, adjusted for age and sex. One of these four studies [55] also adjusted for ethnicity. Additionally, those same four studies [30, 50, 53, 55] adjusted for household income, three of the four [30, 53, 55] adjusted for education, and one [55] adjusted for employment. The two studies [30, 53] that assessed prenatal air pollution exposure also adjusted for parity, maternal smoking and alcohol consumption, and pregnancy period. One study based on the UK Biobank cohort also adjusted for individual-level covariates including smoking, alcohol consumption, physical activity, and BMI [55]. One study [50] that focused on epilepsy in adults also adjusted for pre-existing comorbidities such as stroke, heart disease, hypertension, diabetes, and dyslipidemia. One study [32] focusing on febrile seizures among children controlled for infectious diseases such as viral influenza, gastroenteritis, and exanthem subitum (human herpesvirus 6 infection). Another study [36] based in the contiguous United States, adjusted for county-level urban indicators, population density, median age, median household income, and the proportions of females, Black individuals and White individuals, uninsured adults, children in poverty, unemployed, not graduated from high school among those 25 years of age and older, smokers, adults with obesity, and the number of primary care physicians per 100,000 people.

Case-crossover studies, which involve within-participant comparisons of exposure, inherently control for covariates that do not vary over short periods of time (e.g., race/ethnicity, sex, age, BMI, smoking status, SEP) [61]. Potential confounding by longer-term seasonal trends is also adjusted for by design in case-crossover studies [62, 63]. Time-series regression analyses quantifying short-term effects of environmental exposures are also able to control for time invariant covariates at the population level but require adjustment for time varying covariates such as seasonality and other long-term trends [63, 64]. Among the time-series studies included in this review, two studies on multiple air pollutants [47, 57] adjusted for long term trends, day of the week, and daily Tmean and relative humidity or the average humidex. Another study [59] that focused on O_3_ exposure, adjusted for daily average PM_10_, PM_2.5_, SO_2_, CO, NO_2_, sunlight hours, and rainfall, while also controlling for the day of the week, holidays, and daily Tmean and relative humidity. Among time series studies of ambient temperature exposure, one study [48] adjusted for accumulated precipitation, atmospheric pressure, relative humidity, and number of hours of sunshine; another study [56] adjusted for day of the week, holidays, seasonal and long-term trends, and daily mean relative humidity and PM_2.5_; and another study [35] adjusted only for minimum relative humidity. One time series study on exposures to multiple air pollutants and meteorological variables [29] fit single-pollutant/ meteorological variable models and did not adjust for any other covariates.

Among the 16 air pollution exposure studies, 11 studies adjusted for daily/monthly mean temperature and relative humidity. On the other hand, two of the 12 temperature exposure studies [33, 56] adjusted for exposure to air pollutants and three of the six studies on both air pollution and temperature exposure [34, 42, 43] involved mutual co-exposure adjustment. Of all the studies that involved multiple exposures, only four [31, 38, 49, 52] adjusted for multiple comparisons and none examined potential interactions among air pollutants or between air pollutants and temperature. Exposure to O_3_, a secondary air pollutant generated from photochemical reactions between oxides of nitrogen, volatile organic compounds, and atmospheric oxygen in the presence of sunlight (UV radiation), was assessed in nine studies [28, 29, 39, 43, 47, 49, 51, 57, 59], with effect estimates reported from single-pollutant models while sensitivity testing their robustness to adjusting for NO_2_ and other primary air pollutants in two-pollutant models. Of all the temperature exposure studies, only three [33, 34, 58] adjusted for seasonality. In terms of other environmental covariates, one retrospective cohort study adjusted for road traffic-related and railway noise exposures [30], while another cross-sectional study adjusted for the distance to the nearest major road, traffic intensity on the nearest major road, 24-hour noise pollution, and residential greenspace [55].

### Main Findings

#### Air Pollution

**Fig. 2:**
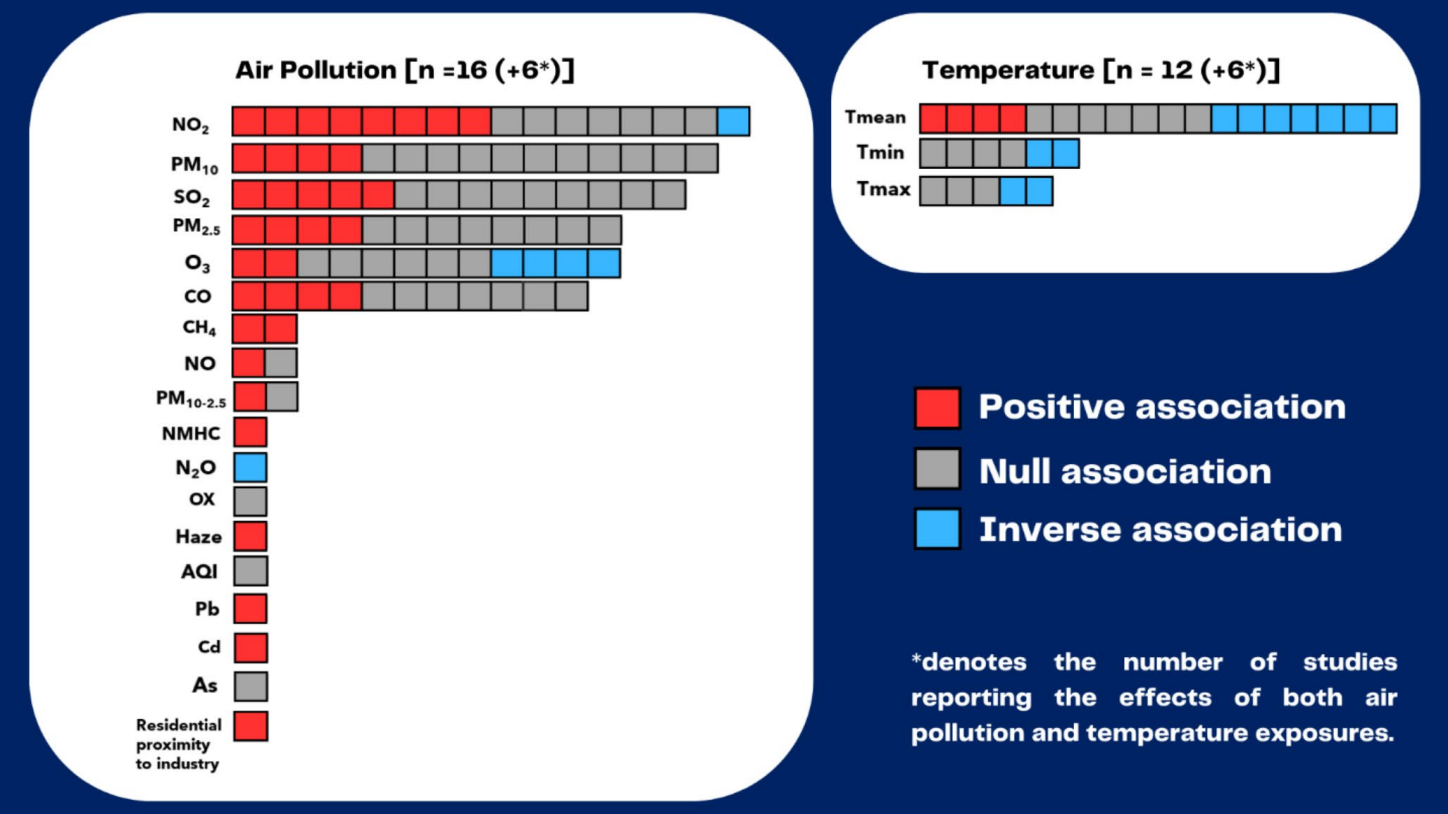
Main findings of studies by exposure; color coded ‘red’ for positive association (harmful effect), ‘gray’ if association included the null, and ‘blue’ for inverse association (protective effect)

Overall, the main findings lacked consistency across studies as mixed results were reported for most of the air pollutants (Fig. 2). For instance, for PM_2.5_, four studies reported positive associations [29, 36, 45, 49] and eight reported associations that included the null [39, 43, 47, 51–53, 55, 57]. For PM_10_, four studies reported positive associations [39, 47, 49, 58] and eleven reported null [28, 29, 34, 42, 43, 45, 51, 52, 54, 55, 57]. For NO_2_, eight studies reported positive associations [29, 30, 45, 47, 49, 51, 55, 57], seven reported null associations [28, 32, 34, 39, 43, 52, 53], and one reported an inverse association [42]. For SO_2_, five studies reported positive associations [39, 43, 49, 57, 58] and nine reported null [28, 29, 32, 34, 42, 45, 51–53]. For CO, four studies reported positive associations [28, 29, 45, 47] and seven reported null [34, 39, 43, 51–53, 57]. For O_3_, a secondary air pollutant, two studies reported positive associations [34, 47], six reported null [28, 29, 39, 43, 49, 53], and four reported an inverse association [34, 51, 57, 59].

Among studies examining the effects of short-term exposures, Bao et al. [45] investigated the relationships between multiple air pollutants and epilepsy-related hospitalization across 10 cities in eastern China. The results of their time-stratified, case-crossover analysis showed that every interquartile (IQR) increase in PM_2.5_, CO, and NO_2_ exposure on the day before hospitalization was associated with 1.32% (95% Confidence Interval (CI): 0.16%, 2.48%), 1.50% (0.3%, 2.6%), and 2.50% (0.6%, 4.3%) excess hospitalization risk, respectively. Additionally, same-day CO and NO_2_ exposures were associated with 1.10% (0.10%, 2.10%) and 2.00% (0.50%, 3.60%) excess risks, respectively. In a similar case-crossover study, Cheng et al. [49] observed that each 10 µg/m^3^ increase in same-day PM_2.5_, and previous day PM_10 − 2.5_, PM_10_, NO_2_, and SO_2_ concentrations were associated with 1.10% (95% CI: 0.10%, 2.10%), 1.70% (0.50%, 2.90%), 0.80% (0.10%, 1.40%), 4.30% (2.30%, 6.30%), and 8.50% (1.50%, 16.0%) increases in childhood epilepsy hospitalizations, respectively, across 10 cities in Anhui Province, China. In a daily time-series analysis, Cakmak et al. [47] demonstrated that air pollution was associated with higher risk of epilepsy hospitalization in Chile. Adjusting for long-term trends, day of the week, and average humidex on the day of hospitalization and the day before, the authors observed elevated hospitalization risks for every IQR increment in exposure to PM_10_ [1.08 (95% CI: 1.04, 1.13)], CO [1.10 (1.05, 1.16)], NO_2_ [1.11 (1.02,1.20)], SO_2_ [1.09 (1.03, 1.14)], and O_3_ [1.10 (1.03, 1.18)]. Additionally, the effect of O_3_ decreased in magnitude but remained statistically significant after adjustment for NO_2_ or PM_10_. In another daily time-series study with a DLNM approach, Kim et al. [34] used medical records to examine the effects of multiple environmental exposures on ED visits for pediatric febrile seizures at a single medical facility in Changwon, South Korea. The authors found inverse as well as positive associations with O_3_ at different a priori selected exposure levels. The cumulative relative risks (across 7 lag days) appeared to increase linearly from inverse associations observed at the 1st percentile (0.005 ppm), 25th percentile (0.019 ppm), and 50th percentile (0.026 ppm) [cumulative RR: 0.51 (95% CI: 0.43, 0.61), 0.77 (0.72, 0.83), 0.94 (0.93, 0.96), respectively, vs. the median exposure level (not reported)] to positive associations observed at the 75th percentile (0.035 ppm) and 99th percentile (0.064 ppm) [1.23 (1.16, 1.29) and 2.84 (2.14, 3.77), respectively].

Among studies examining the effects of long-term exposures, Ronaldson et al. [55] in their cross-sectional assessment based out of the UK Biobank cohort, reported a null association between annual average NO_2_ exposure and epilepsy diagnosis [Odds Ratio (OR):1.22 (95% CI: 0.98, 1.51) for exposure quartile 4 (> 31.22 µg/m^3^) vs. quartile 1(< 21.32 µg/m^3^)]. Min et al. [36] reported a 1 µg/m^3^ increase in lag 0–1 years PM_2.5_ exposure was positively associated with an increased risk of convulsions [RR: 1.04 (95% Credible Interval: 1.01, 1.06)] across New York State, USA. In a Danish retrospective cohort study, Hjortebjerg et al. [30] examined how the average NO_2_ exposure during pregnancy and yearly, post-natal NO_2_ exposure at the time of diagnosis, estimated at every residential address of each participant, were related to the occurrence of febrile seizures in children under the age of 6 years during a 7-year follow-up period. The authors found a positive association with childhood exposures after adjusting for various maternal covariates; every IQR increase in NO_2_ exposure was associated with an incidence rate ratio (IRR) of 1.05 (95% CI: 1.02, 1.07). However, the effect was attenuated when further adjustment with co-exposure to road traffic noise was made [IRR: 1.03 (95% CI: 0.99, 1.06)]. In another retrospective cohort, Lee et al. [53] assessed the impact of elevated concentrations of multiple air pollutants and heavy metal exposures during pregnancy on the incidence of epilepsy, using South Korea’s National Health Insurance claims data. The authors, who did not adjust for multiple comparisons, found that higher levels of Pb exposure during the first trimester of pregnancy and Cd exposure during the third trimester were associated with increased odds of epilepsy [OR:1.11 (95% CI:1.04, 1.18) and 2.19 (1.07, 4.48), respectively].

Some studies have reported stronger subgroup effects among children, older adults, and females. For example, Bao et al. [45] reported stronger NO_2_ and CO effects among children aged < 18 years but observed no variation by sex. Xu et al. [57] reported stronger NO_2_ and SO_2_ effects among children aged < 18 years and O_3_ effects among adults aged 18–59 years. They also reported stronger effects of NO_2_ in females and SO_2_ and O_3_ effects in males. Chen et al. [28] observed increased seizure risk among females with CO and NO_2_ exposures compared to males. Stronger, but protective, O_3_ effects were reported by Zhou et al. [59] in children aged < 18 years and older adults aged > 65 years, and females. The protective O_3_ effects remained robust after adjustment for PM_10_, PM_2.5_, SO_2_, CO, and NO_2_.

Farahmandfard et al. [51] observed stronger associations of epilepsy-related hospital admissions in Kerman, Iran, with NO_2_ and CO exposures on lag day 0 among older adults aged > 59 years and CO on lag day 4 among males. Choi et al. [50] observed stronger associations of epilepsy incidence with residential proximity to industrial complexes in South Korea among older adults aged > 65 years, females, and those without preexisting diabetes. Hjortebjerg et al. [30] observed an interaction of NO_2_ effects with maternal parity; stronger effects were observed for uniparous (1 previous birth) and multiparous births (> 2 previous births) in a dose-response manner vs nulliparous birth (first child). Min et al. [36] observed stronger PM_2.5_ effects among females and racial minorities [classified as ‘Other’ races (American Indian/ Alaska Native, Asian, and Native Hawaiian/Pacific Islander) in New York State]. Some studies reported seasonal variations in the effects of certain pollutants. Cheng et al. [49] reported stronger PM_2.5_, PM_10_, and PM_10 − 2.5_ effects in the cold season. Stronger but protective cold season effects were also reported for O_3_ by Zhou et al. [59]. Cakmak et al. [47] observed no variations by age, sex, or season.

#### Ambient Temperature

Overall, the main findings for ambient temperature lacked consistency across studies as mixed results were reported (Fig. 2). Among the studies that used Tmean as the exposure metric, four reported positive associations [29, 41, 44, 58], seven reported associations that included the null [31, 32, 34, 38, 40, 42, 43], and seven reported inverse associations [27, 33, 37, 41, 46, 48, 56]. For Tmin, four studies reported null [33, 40, 43, 46] and two studies reported inverse associations [27, 35]. For Tmax, three studies reported null [33, 40, 43] and two studies reported inverse associations [27, 35].

In a monthly time-series analysis, Chang et al. [48] utilized the national health insurance claims database to explore the associations between multiple meteorological factors and epilepsy-related ED visits in Taiwan. Following mutual co-exposure adjustments between accumulated precipitation, atmospheric pressure, relative humidity, and number of hours of sunshine, lower temperature, defined on a continuous scale, emerged as the sole influential meteorological factor. Specifically, a 1 °C *decrease* in Tmean was associated with a relative risk (RR) increase of 1.02 (95% CI: 1.01, 1.02). Moreover, monthly Tmean below 18 °C were identified to have the highest predictive value for epileptic seizure events. In a daily time-series analysis, adjusting for the day of the week, holidays, seasonal and long-term trends, relative humidity, and PM_2.5_ exposures, Sun et al. [56] examined the relationship of daily apparent temperature (AT; calculated from Tmean, relative humidity, and wind velocity) with epilepsy-related clinic visits in Hefei, China. Using the DLNM framework, the authors found positive associations across five lag days with *low* AT, whereas high AT did not show similar effects. The strongest effect of low AT [defined at the 5th percentile (-1.5 °C) vs. the median (17 °C)] on the clinic visits occurred on the lag day 1 [Relative Risk (RR):1.06 (95% CI:1.02, 1.10)], followed by lag 2 [1.05 (1.02, 1.08)], lag 3 [1.05 (1.02, 1.07)], lag 4 [1.04 (1.02, 1.07)], and lag 5 [1.04 (1.01, 1.07)].

Utilizing the national health insurance claims database in Taiwan, Chiang et al. [29] conducted a univariable, daily time-series analysis between temperature exposures and hospital visits for epileptic seizures. The authors found a 6.37% (95: CI: 4.40%, 8.34%) increase in the number of hospital visits per 10 °C increase in cumulative 7-day lag Tmean exposures. In another daily time-series analysis, adjusting for seasonality, long-term trend, day of week, and public holidays, Yalçın et al. [58] quantified the relationship of temperature with ED visits for pediatric epilepsy at a children’s hospital in Diyarbakır, Turkey. The authors found an Incident Rate Ratio of 1.03 (95% CI: 1.01, 1.06) per 1 °C increase in daily Tmean. In Kaiserslautern, Germany, Zhang et al. [44] conducted a time-stratified, case-crossover analysis within the DLNM framework to examine the relationship between temperature and hospitalization for epileptic seizures during the hot season in Brazil, covering nearly 79% of the country’s population. Each 1 °C increase in the cumulative 7-day lag Tmean, above the impact threshold (26 °C), was linked to an Odds Ratio of 1.04 (95% CI: 1.03, 1.06) for the risk of hospitalization. Across the seven lag days, the strongest effect was observed on lag day 1 and decreased non-linearly up to lag day 7.

In a study in Seoul, South Korea, Woo et al. [41] observed that the risk of first hospital visits for febrile seizures among children increased bimodally with Tmean in a lower temperature range (-7 °C to -1 °C) and at a higher temperature range (18 °C to 21 °C) [effect estimates not reported]. Kim et al. [33] used DLNM functions to test for non-linear Tmean effects on four types of pediatric seizures in Changwon, South Korea. The authors found positive associations at lower Tmean extremes (defined a priori) and inverse associations at higher Tmean extremes (defined a priori). The cumulative relative risk across 15 lag days for Tmean appeared to decrease linearly from positive associations observed at the 0.1th percentile (-4.8 °C) and the 5th percentile (0.4 °C) vs. the median (15.9 °C) [cumulative RR: 1.66 (95% CI: 1.04, 2.66) and 1.46 (1.03, 2.08), respectively] to inverse associations observed at the 95th percentile (27.4 °C) and the 99.9th percentile (30.1 °C) vs. the median (15.9 °C) [0.75 (0.58, 0.98) and 0.71 (0.51, 0.97), respectively]. However, in another similar study that focused exclusively on pediatric febrile seizures in Changwon, South Korea, Kim et al. [34] did not observe effects of Tmean.

Among studies reporting sub-group effects of temperature exposures, Sun et al. [56] observed stronger effects of lower apparent temperatures on epileptic seizures among younger age groups (0–14 years and 15–29 years) in Hefei, China, and Zhang et al. [44] observed stronger effects of high Tmean exposures on epileptic seizures among females, individuals aged 20–39 years, and persons living in high-income regions in Brazil.

## Discussion

Understanding the role of air pollution and temperature exposure in determining the risk of seizures and epilepsy – particularly given the high proportion of unknown etiology – is an emerging issue in environmental epidemiology, and several studies have been published in the last two decades. The etiology of incidence as well as acute aggravation of seizures and epilepsy remains largely unknown, and identifying their potentially modifiable risk factors can prove instrumental in reducing the substantial public health and economic burden associated with these conditions [65]. In this review, overall, the main findings across studies lacked consistency as mixed results were reported for all air pollutants and temperature metrics of interest. This may be attributable to differences in sample sizes, outcomes, exposure assessments, analytical methods, and underlying biases. Here, we highlight potential limitations of the reviewed studies along with corresponding opportunities for future research.

Bias due to exposure misclassification is a potential concern. Studies included in this review mostly assigned area (city or county)-level ambient exposure estimates to seizures and epilepsy cases presenting to an ED. Personal exposure may substantially differ from ambient measurements taken, for instance, from a stationary monitor because personal exposure may vary according to the time, location, and activity of each individual, even in the short term [66]. None of the studies measured personal exposures but would need to be (presumptively) very highly correlated with the ambient measurements to plausibly explain the reported relationships. Exposure misclassification may also occur due to residential and occupational mobility, particularly in studies of chronic exposure [67, 68]. Among the long-term exposure studies included in this review, only one study [30] accounted for residential mobility by collecting residential histories of all participants and assigning exposures at each residential address. The other studies are prone to non-differential exposure misclassification at least to some extent and, if present, it may have biased the results (likely but not always) toward the null [66, 69, 70]. Future studies may assess personal exposures considering time-location-activity profiles, including residential and occupational mobility, to minimize exposure measurement errors. Indoor air pollution and temperature [71, 72] have also been linked to adverse neurological outcomes and, therefore, may be assessed for their epileptogenic potential. Quantifying the effects of chemical constituents of particulate matter pollution [73] and intensifying climate-linked exposures such as wildfire smoke [74] are other research avenues.

In studies examining effects of multiple exposures involving co-exposure adjustments, inflation of type I (α) error due to multiple comparisons may yield uncontrolled false-positive test results. Only four studies [31, 38, 49, 52] included in this review adjusted for multiple comparisons using the Bonferroni correction method. Future studies assessing the impact of multiple exposures should correct for multiple comparisons/ multiple testing using Bonferroni-related or other available methods [75]. Additionally, none of the studies reviewed examined interactions among air pollutants and between air pollutants and temperatures. Several studies have demonstrated their joint effects on respiratory and cardiovascular morbidity and mortality [76] and should be explored in the context of seizures, epilepsy, and other neurocognitive outcomes. Advanced analytical environmental exposure mixture methods may help shed light on the health effects of real-life exposure scenarios, where individuals and populations are often exposed to diverse mixtures of chemical and non-chemical exposures and not to a single chemical in isolation [77, 78]. Furthermore, as demonstrated by a handful of the studies included in this review [33, 46], associations with ambient temperature may vary depending on the choice of temperature metric used. Future studies may compare and contrast findings when using different temperature metrics, including diurnal temperature ranges and different heat wave/cold spell/ extreme temperature definitions [79–81].

Outcome misclassification is also likely to occur. Nearly all studies reviewed relied on hospital admissions, ED visits, or insurance claims data for passive outcome ascertainment. Errors and inaccuracies in linking such data may result in biases related to misclassification, especially when the variables used for linkage are of subpar quality [82–84] and if present, such misclassification may bias the results toward the null [84]. Additionally, the processes of recording and registering administrative healthcare data, which establish populations and outcomes, can introduce selection bias [84]. A systematic review and meta-analysis of studies evaluating the diagnostic accuracy of administrative healthcare datasets in identifying epilepsy cases reported that for studies using medical records as the diagnostic gold standard the positive predictive value (PPV) ranged from 5.2 to 100%, negative predictive value (NPV) ranged from 13.2 to 100%, and sensitivity, as well as specificity, ranged 61.0–100% [85]. The meta-analytic findings indicated high PPV and sensitivity (estimates exceeding 80%) across diverse coding systems and algorithms, such as ICD-8, ICD-9, ICD-10, and Read Codes irrespective of the presence or absence of antiepileptic drugs (AEDs) or procedures. These results were consistent across different age groups and healthcare settings, including accident and emergency, inpatient, outpatient, and primary care [85]. Longitudinal studies with active case ascertainment may help correctly and timely identify seizures and epilepsy cases for clinical management purposes [86, 87]. However, considering the low prevalence of these disorders at the population scale [8], longitudinal studies may get resource-intensive and administrative healthcare data could still prove useful in identifying people with epilepsy for epidemiological research purposes [85].

Future studies using administrative healthcare data for outcome ascertainment, as recommended by Mbizvo et al. [85], may focus on combining disease codes with information on AED use and should refrain from using AEDs data alone for identifying persons with seizures and epilepsy. Also, researchers should be cognizant of the recognized trade-off relationship between PPV and sensitivity [88]. In situations where researchers aim to emphasize achieving a high PPV, Mbizvo et al. [85] propose the inclusion of disease codes (ICD-10 G40-41, ICD-9 345) without symptom codes (ICD-10 R56, ICD-9 780.3, 780.39), along with one or more AEDs. In scenarios where a balance is sought between PPV and sensitivity, researchers might opt for disease codes alone (excluding AEDs). Alternatively, when sensitivity takes precedence, it could be beneficial to incorporate symptom codes along with one or more AEDs. Further, as the use of AEDs could modify subsequent responsivity to air pollution or temperature exposures, or alter treatment strategies in such a way as to shift encounters from the ED to private providers; future studies may account for this effect by focusing exclusively on or performing a subgroup analysis on the first seizure/epilepsy diagnoses, as done under some studies reviewed [27, 30, 32]. Contingent upon adequate sample size, future studies should also consider assessing potential effect modification (e.g., through stratification) by different subtypes of seizures and epilepsies to help delineate the underlying etiologic and mechanistic pathways linking the environmental exposures with outcomes. Studies included in this review often combined different subtypes of seizures or epilepsy outcomes into a single group—a decision that might have been driven by the motivation to achieve adequate statistical power, although was not explicitly mentioned in any of the studies. Only one study [33] included in this review assessed subgroup effects by seizure subtypes and found stronger associations between lower temperature extremes and pediatric febrile seizure ED visit risk in Changwon, China, and not with afebrile seizures, seizures of known epilepsy, and status epilepticus.

Confounding and/or effect modification by individual- and community-level indicators of socioeconomic position (SEP) is another concern, especially among studies that use administrative healthcare data largely designed to collect information on service utilization and associated costs. Chronic stress that may be conferred by various stressors constituting the SEP, has been linked with increased seizure and epilepsy risk [89, 90]. Lower SEP communities are often exposed to elevated levels of air pollution and temperature extremes [91–95] and bear a greater burden of seizures and epilepsy than socioeconomically advantaged groups [96, 97]. In addition to case-crossover studies that adjust for time invariant covariates by design, only four studies included in this review [30, 50, 53, 55] adjusted for individual-level SEP-*related* variables such as household income [30, 50, 53, 55], education [30, 53, 55], and employment [55], and only one study [44] performed subgroup analysis by municipality-level income and another by country-level racial/ethnic composition [36]. Cognizant of the distinctions between “context” (the inherent characteristics of a place) and “composition” (the attributes of the people residing in that area), future studies should account for confounding by SEP [98] and explore interactions among individual stressors (such as work-related strain and victimization), social stressors that affect entire communities (such as poverty, violent crime, and structural racism), and air pollution and temperature exposures that are often concentrated within groups of individuals in the same communities [99–102].

Similarly, potential confounding by other meteorological factors such as atmospheric pressure and humidity, which were also found to be associated with seizures and epilepsy in some of the reviewed studies [27, 31, 35, 37, 41], may also be adjusted for. However, the resulting main effects, particularly in the case of temperature, after such confounder adjustment should be interpreted with caution [103, 104]. Humidity can influence temperature and adjusting for humidity can deliberately remove the influence of humidity on both temperature and the health outcome, yielding controlled direct effect of temperature (and not the total effect) [103, 104]. Moreover, temperature and humidity can be highly correlated and including both variables in a multivariable regression model can lead to challenges related to multicollinearity—more complexly so when the correlation might be non-linear or time dependent—that may decrease the precision of the observed effects of temperature (or humidity) [103]. Alternatively, using an interaction term between humidity and temperature to represent potential effect modification or interaction (explored on both additive and multiplicative scales) can be considered [103]. While the analytical methods for assessing interaction or effect modification (such as interaction terms or stratification) are comparable, the policy implications may vary when the focus is on addressing exposure to temperature, humidity, or both simultaneously [103]. Furthermore, while several studies included in this review adjusted for the potential effects of relative humidity, future studies may consider mass-based measures (e.g., specific or absolute humidity) over relative humidity when choosing a humidity variable [103]. Mass-based measures directly affect the evaporation of sweat, whereas relative humidity demonstrates daily temperature-dependent patterns that do not accurately represent changes in heat stress [103].

Of all studies on temperature exposure included in this review, only three [33, 34, 58] adjusted for seasonality of various meteorological conditions. Case-crossover studies, by design, do not require to adjust for seasonality [62, 63]. Time series regressions, however, should adjust for seasonality and other long-term trends to accurately explain short-term variations in the outcome (here, for example, seizures and epilepsy related morbidity) that can be attributed to temperature exposure [63]. In addition to meteorology, seasonality can be related to other factors that show seasonal variations such as influenza, Vitamin D levels, and socio-behavioral phenomena [63, 105], which might confound associations. For example, associations observed between low ambient temperatures and seizures/ epilepsy may be confounded by winter seasonality of febrile upper respiratory tract illnesses [106] or low Vitamin D levels from reduced sunlight exposure [107].

Finally, future investigators examining temperature effects, particularly over the short term, should provide a clear rationale for their decision to adjust or not to adjust for potential air pollution effects. Some temperature studies included in this review included air pollutants as covariates in their analyses, thereby reporting temperature effects adjusted for air pollution. However, this practice of adjusting for air pollutants while estimating temperature effects, without explicit mention of what effects are being estimated may not be appropriate and requires careful consideration as it impacts the interpretation of findings vis-à-vis their policy implications [108]. For instance, if the pollutant of interest (e.g., ozone) is on the pathway between temperature and the outcome, then it does not meet the criteria for being a confounder of the associations. If ozone, in this example is adjusted for, one would be adjusting away any effects of temperature on the outcome that operate through the air pollutant. The resulting temperature effect, therefore, would be a direct effect and not a total effect on the outcome. None of the studies reviewed made explicit distinctions between the estimation of direct versus total effects of temperature when adjusting for air pollutants. Future studies should make these distinctions clear and consider the use of Directed Acyclic Graphs or DAGs in evaluating the causal structures between temperature, air pollution, and health outcome(s). This is discussed in detail by Buckley et al. [108].

This review per se had some limitations. We did not include studies that might have been published in languages other than English, animal studies, and experimental studies which may add unique insights. While we provided summaries and interpretations of the included studies, which is consistent with a scoping review, it is important to note that our approach, although structured, was not entirely that of a systematic review. We did not evaluate the overall strength of the evidence by considering factors such as sample size, study design, or potential biases commonly assessed to gauge the study quality. Moreover, studies included in this review were heterogeneous in terms of how exposures and outcomes were ascertained and therefore, a more formal systematic review or meta-analysis could not be conducted [109]. Nevertheless, our extensive review yielded a substantial amount of valuable information concerning the scope of existing literature. Contingent upon sufficient numbers of homogeneous studies with comparable exposure and outcome ascertainment, logical progression would involve conducting a systematic review or meta-analysis to answer specific research questions [109]. Our review’s findings could be also influenced by publication bias, as studies that are published are more inclined to highlight statistically significant and positive associations, potentially impacting the results [110]. Future systematic reviews and meta-analyses should assess for any publication biases using tests for the asymmetry of funnel plots and/or methods based on selection models or weighted distributions [110].

## Conclusion

An increasing body of epidemiological evidence indicates that exposure to air pollution and temperature extremes may be associated with seizures and epilepsy outcomes. Further research is needed to address the limitations of existing studies and fill identified knowledge gaps to reach definitive causal conclusions and support environmental policies and actions aimed at addressing the disproportionate health impacts of air pollution and temperature-related exposures, which are likely to worsen with progressing climate change.

## Electronic Supplementary Material

Below is the link to the electronic supplementary material.

Supplementary Material 1

## Data Availability

No datasets were generated or analysed during the current study.
