# Supplementary material for: Air Pollution and Temperature in Seizures and Epilepsy: A Scoping Review of Epidemiological Studies
Source: Curr Environ Health Rep. 2024 Dec 10;12(1):1. doi: 10.1007/s40572-024-00466-3 (PMC11631820; doi:10.1007/s40572-024-00466-3)
Supplement: Supplementary file 1 — Supplementary Material 1 [file 40572_2024_466_MOESM1_ESM.docx]

# Supplementary Materials

**Abbreviations and symbols:**

PM_2.5_, particulate matter less than 2.5 μm in aerodynamic diameter

PM_10_, particulate matter less than 10 μm in aerodynamic diameter

PM_10-2.5_ or PMcoarse, particulate matter with aerodynamic diameter between 2.5 μm and 10 μm

NO_2_, nitrogen dioxide

NO, nitric oxide

N_2_O, nitrous oxide

NMHC, non-methane hydrocarbons

CH_4_, methane

CO, carbon monoxide

SO_2_, sulfur dioxide

O_3_, ozone

OX, oxidant

VOCs, Volatile Organic Compounds

PAHs, Polycyclic Aromatic Hydrocarbons

Pb, Lead

Cd, Cadmium

Tmean, Mean temperature

Tmin, Minimum temperature

Tmax, Maximum temperature

DTR, Diurnal Temperature Range

RH, Relative humidity

°F: degree Fahrenheit

°C degree Celsius

μg/m³, microgram per cubic meter

ppb, parts per billion

ppm, parts per million

ppmC, concentration unit of parts per million carbon

hPa, hectopascal

ICD-9, the International Classification of Diseases, 9th version

ICD-10, the International Classification of Diseases, 10th version

SUDEP, Sudden Unexpected Death in Epilepsy

RR, Relative Risk

OR, Odds Ratio

IRR, Incident Rate Ratio

HR, Hazard Ratio

95% CI, 95% Confidence Interval

SE, Standard Error

IQR, Interquartile Range

NA, Not Available

WHO, World Health Organization

↑, increase

↓, decrease

**Table S1: Search Strategy**

| **Database** | | **Search terms** |
| --- | --- | --- |
| Ovid MEDLINE | | |
| 1 | Exposures (air pollution and temperature) | **exp Air Pollution/ or exp Air Pollutants/ or exp Particulate Matter/ or exp Weather/ or exp Climate/ or exp Climate Change/ or exp Meteorology/** OR air pollut$.tw. OR airborne partic$.tw. OR airborne pollut$.tw. OR traffic pollut$.tw. OR air qualit$.tw. OR air toxic$.tw. OR ultrafine partic$.tw. OR total suspended partic$.tw. OR Ozone.tw. OR O3.tw. OR Carbon Monoxide.tw. OR Nitrogen Dioxide.tw. OR NO2.tw. OR Nitrogen Oxide$.tw. OR Nitric Oxide.tw. OR Nitrous Oxide.tw. OR Sulfur Dioxide.tw. OR Sulphur Dioxide.tw. OR SO2.tw. OR Particulate Matter$.tw. OR PM25.tw. OR PM10.tw. OR Smog.tw. OR Soot.tw. OR Smoke.tw. OR Dust.tw. OR Black Carbon.tw. OR Elemental Carbon.tw. OR climate change.tw. OR weather$.tw. OR hot temperature$.tw. OR cold temperature$.tw. OR meteorolog$.tw. |
| 2 | Outcomes (seizures and epilepsy) | **exp Seizures, Febrile/ OR exp Seizures/ OR exp Psychogenic Nonepileptic Seizures/ OR exp epilepsy/ OR exp drug resistant epilepsy/ OR exp epilepsies, partial/ OR exp epilepsy, benign neonatal/ OR exp epilepsy, generalized/ OR exp epilepsy, reflex/ OR exp epileptic syndromes/ OR exp sudden unexpected death in epilepsy/** OR seizure$.tw. OR convuls$.tw. OR epilep$.tw. |
| Search query | | 1 AND 2 |
| Embase | | |
| 1 | Exposures (air pollution and temperature) | **('air pollution'/exp OR 'particulate matter'/exp OR 'weather'/exp OR 'climate change'/exp)** OR (((“air pollut$” OR “airborne partic$" OR “airborne pollut$” OR “traffic pollut$” OR “air qualit$” OR “air toxic$” OR “ultrafine partic$” OR “total suspended partic$” OR Ozone OR O3 OR 'carbon monoxide' OR 'nitrogen dioxide' OR 'NO2' OR “nitrogen oxide$” OR 'nitric oxide' OR 'nitrous oxide' OR 'sulfur dioxide' OR 'sulphur dioxide' OR SO2 OR “particulate matter$” OR PM25 OR PM10 OR smog OR soot OR smoke OR dust OR black) AND carbon OR elemental) AND carbon OR 'climate change' OR weather$ OR “hot temperature$” OR “cold temperature$” OR meteorolog$):ab,ti,kw |
| 2 | Outcomes (seizures and epilepsy) | **'seizure'/exp OR 'epilepsy'/exp** OR seizure$:ab,ti,kw OR convuls$:ab,ti,kw OR epilep$:ab,ti,kw |
| Search query | | 1 AND 2 |
| APA PsycINFO | | |
| 1 | Exposures (air pollution and temperature) | **(MM "Pollution") OR (DE "Atmospheric Conditions" OR DE "Climate Change" OR DE "Extreme Weather")** OR air pollut$ OR airborne partic$ OR airborne pollut$ OR traffic pollut$ OR air qualit$ OR air toxic$ OR ultrafine partic$ OR total suspended partic$ OR Ozone OR O3 OR Carbon Monoxide OR Nitrogen Dioxide OR NO2 OR Nitrogen Oxide$ OR Nitric Oxide OR Nitrous Oxide OR Sulfur Dioxide OR Sulphur Dioxide OR SO2 OR Particulate Matter$ OR PM25 OR PM10 OR Smog OR Soot OR Smoke OR Dust OR Black Carbon OR Elemental Carbon OR climate change OR weather$ OR hot temperature$ OR cold temperature$ OR meteorolog$ |
| 2 | Outcomes (seizures and epilepsy) | **DE "Seizures" OR DE "Audiogenic Seizures" OR DE "Epileptic Seizures" OR DE "Grand Mal Seizures" OR DE "Petit Mal Seizures" OR DE "Status Epilepticus" OR DE "Epileptic Seizures" OR DE "Experimental Epilepsy" OR DE "Epilepsy" OR DE "Epileptic Seizures" OR DE "Experimental Epilepsy" OR DE "Lennox Gastaut Syndrome"** OR seizure$ OR convuls$ OR epilep$ |
| Search query | | 1 AND 2 |
| Web of Science | | |
| 1 | Exposures (air pollution and temperature) | **TI=('air pollution'/exp OR 'particulate matter'/exp OR 'weather'/exp OR 'climate change'/exp)** OR TI=(“air pollut$” OR “airborne partic$" OR “airborne pollut$” OR “traffic pollut$” OR “air qualit$” OR “air toxic$” OR “ultrafine partic$” OR “total suspended partic$” OR Ozone OR O3 OR 'carbon monoxide' OR 'nitrogen dioxide' OR 'NO2' OR “nitrogen oxide$” OR 'nitric oxide' OR 'nitrous oxide' OR 'sulfur dioxide' OR 'sulphur dioxide' OR SO2 OR “particulate matter$” OR PM25 OR PM10 OR smog OR soot OR smoke OR dust OR ‘black carbon’ OR 'climate change' OR weather$ OR “hot temperature$” OR “cold temperature$” OR meteorolog$) OR **AB=('air pollution'/exp OR 'particulate matter'/exp OR 'weather'/exp OR 'climate change'/exp)** OR AB=(“air pollut$” OR “airborne partic$" OR “airborne pollut$” OR “traffic pollut$” OR “air qualit$” OR “air toxic$” OR “ultrafine partic$” OR “total suspended partic$” OR Ozone OR O3 OR 'carbon monoxide' OR 'nitrogen dioxide' OR 'NO2' OR “nitrogen oxide$” OR 'nitric oxide' OR 'nitrous oxide' OR 'sulfur dioxide' OR 'sulphur dioxide' OR SO2 OR “particulate matter$” OR PM25 OR PM10 OR smog OR soot OR smoke OR dust OR ‘black carbon’ OR 'climate change' OR weather$ OR “hot temperature$” OR “cold temperature$” OR meteorolog$) OR **AK=('air pollution'/exp OR 'particulate matter'/exp OR 'weather'/exp OR 'climate change'/exp)** OR AK=(“air pollut$” OR “airborne partic$" OR “airborne pollut$” OR “traffic pollut$” OR “air qualit$” OR “air toxic$” OR “ultrafine partic$” OR “total suspended partic$” OR Ozone OR O3 OR 'carbon monoxide' OR 'nitrogen dioxide' OR 'NO2' OR “nitrogen oxide$” OR 'nitric oxide' OR 'nitrous oxide' OR 'sulfur dioxide' OR 'sulphur dioxide' OR SO2 OR “particulate matter$” OR PM25 OR PM10 OR smog OR soot OR smoke OR dust OR ‘black carbon’ OR 'climate change' OR weather$ OR “hot temperature$” OR “cold temperature$” OR meteorolog$) |
| 2 | Outcomes (seizures and epilepsy) | **TI=('seizure'/exp OR 'epilepsy'/exp)** OR TI=(seizure$ OR convuls$.tw. OR epilep$) OR **AB=('seizure'/exp OR 'epilepsy'/exp)** OR AB=(seizure$ OR convuls$.tw. OR epilep$) OR **AK=('seizure'/exp OR 'epilepsy'/exp)** OR AK=(seizure$ OR convuls$.tw. OR epilep$) |
| Search query | | 1 AND 2 |

**Table S2: Summary of studies examining relationships between air pollution exposure and seizures and epilepsy.**

| **First author and publication year** | **Study location and duration** | **Study design and analytical approach** | **Study population and outcome(s)** | **Exposure(s)** | **Exposure resolution and assessment method** | **Covariate(s)** | **Main findings** | **Other findings/comment(s)** |
| --- | --- | --- | --- | --- | --- | --- | --- | --- |
| Bao, 2019 [45] | Eastern China, 2014-2015. | Time-stratified case-crossover; conditional Poisson regression. | 51,523 epilepsy cases per ICD-10 classification (codes G40 and G41) presented at 47 tertiary care hospitals across 10 cities. | PM_2.5_, PM_10_, NO_2_, SO_2_, and CO. | Daily, citywide mean concentrations from ground monitoring stations under the National Air Pollution System. | Daily Tmean and RH. | *Percent excess risk (95% CI) per IQR increase in exposures (overall results):* ▪Lag 0 NO_2_: 2.00% (0.50%, 3.60%) per 25.9 µg/m^3^ ↑  ▪Lag 1 NO_2_: 2.50% (0.60%, 4.30%) per 25.9 µg/m^3^ ↑  ▪Lag 0 CO: 1.10% (0.10%, 2.10%) per 0.50 µg/m^3^ ↑ ▪Lag 1 CO: 1.50% (0.30%, 2.60%) per 0.50 µg/m^3^ ↑  ▪Lag 1 PM_2.5_: 1.32% (0.16%, 2.48%) per 56.9 µg/m^3^ ↑ | ▪Stronger effects in children and adolescents (age <18 years). ▪No variation by sex. ▪Sensitivity-tested 2-pollutant models. Same-day CO and NO_2_ effects were stronger when adjusted for PM_2.5_ and PM_10._ |
| Cakmak, 2010 [47] | Chile, 2001-2005. | Daily time-series; Poisson regression and random effects pooled analysis | 290,500 epilepsy cases, per ICD-10 classification (codes G40 and G41), presented at hospitals across 7 cities. | PM_2.5_, PM_10_, NO_2_, SO_2_, CO, and O_3._ | Daily, citywide mean concentrations from 7 ground monitoring stations across the 7 urban centers. | Long term trends, day of the week, and average humidex on the day of hospitalization and the day before. | *RR (95% CI) of hospitalization per IQR increase in exposures (pooled results):* ▪PM_2.5_: 1.07 (1.00, 1.13) per 21.51 µg/m^3^ ↑ ▪PM_10_: 1.08 (1.04, 1.13) per 37.79 µg/m^3^ ↑  ▪NO_2_: 1.11 (1.02, 1.20) per 28.97 ppb ↑  ▪SO_2_: 1.09 ( 1.03, 1.14) per 6.20 ppb ↑ ▪O_3_: 1.10 (1.03, 1.18) per 69.51 ppb↑  ▪CO: 1.10 (1.05, 1.16) per 1.15 ppm↑ | ▪No variation by age, sex, or season. ▪Sensitivity-tested 2-pollutant models. Only O_3_ statistically significant after adjustment for NO_2_ or PM_10_. PM_10_ remained statistically significant only with adjustment for O_3_, but not other pollutants. |
| Chen, 2022 [28] | Australia, 2010-2012 and 2018-2021. | Time-stratified case-crossover; conditional Poisson regression. | 6692 seizure events (from 49 epilepsy patients) identified using long-term intracranial electroencephalography (iEEG) and a seizure diary mobile application. | PM_10_, NO_2_, SO_2_, O_3_, and CO. | Daily, nearest monitor mean concentrations from ground monitoring stations maintained by the Australian Environment Protection Authority. | Daily Tmean, RH, sun radiation, and precipitation. | *RR (95% CI) of seizure event per IQR increase in exposure (overall results):* ▪CO: 1.04 (1.01, 1.07) per 0.13 ppm ↑  ▪NO_2_: 1.04 (0.98, 1.10) per 6.55 ppb ↑  ▪O_3_: 0.99 (0.94, 1.05) per 9.99 ppb ↑  ▪PM_10_: 1.00 (0.97, 1.03) per 9.60 µg/m^3^ ↑  ▪SO_2_: 0.98 (0.95, 1.00) per 0.60 ppb ↑ | ▪Observed increased seizure risk among females with CO and NO_2_ exposures. ▪Also observed increased risk of subclinical seizures with CO exposure. ▪Sensitivity tested two-pollutant models. CO effects remained robust; SO_2_ after CO or NO_2_ adjustment showed inverse association. |
| Cheng, 2022 [49] | Anhui, China, 2016-2018. | Time-stratified case-crossover; conditional logistic regression and random effects meta-analysis. | 8,181 childhood epilepsy cases per ICD-10 classification (codes G40 and G41) presented at 18 hospitals across 10 cities. | PM_2.5_, PM_10-2.5_, PM_10_, NO_2_, SO_2_, O_3._ | Daily, citywide mean concentrations from 46 ground monitoring stations under the National Urban Air Quality Realtime Publishing Platform. | Daily Tmean and rainfall. | *Percent excess hospitalization risk (95% CI) per 10-unit increase in exposures (overall results):* ▪Lag 0 PM_2.5_: 1.10% (0.10%, 2.10%) per 10 µg/m^3^ ↑  ▪Lag 1 PM_10-2.5_: 1.70% (0.50%, 2.90%) per 10 µg/m^3^ ↑  ▪Lag 1 PM_10_: 0.80% (0.10%, 1.40%) per 10 µg/m^3^ ↑ ▪Lag 1 NO_2_: 4.30% (2.30%, 6.30%) per 10 µg/m^3^ ↑  ▪Lag 1 SO_2_: 8.50% (1.50%, 16.0%) per 10 µg/m^3^ ↑ | ▪Focused only on children (=<18 years age). ▪Observed stronger PM effects in cold season. ▪Observed no variation by age or gender ▪Sensitivity tested two-pollutant models. NO_2_ and O_3_ effects remained robust; others were modified. |
| **Chiang, 2021** [29] | Taiwan, 2009-2013. | Daily time-series; Poisson regression. | 1,010,027 epileptic seizure events (from 180,175 epilepsy cases), per ICD-9 classification (codes 345 and 780.39), identified from the Bureau of National Health Insurance (NHI) claim database. | PM_2.5_, PM_10_, NO, NO_2_, SO_2_, O_3_, CO, CH_4_, NMHC, Tmean, maximum temperature difference, rainfall, RH, atmospheric pressure, wind speed, Ultraviolet B, pH scale of rain, electric conductivity of rain. | Daily, citywide mean concentrations from 77 air quality ground monitoring stations from the Taiwan Air Quality Monitoring Database and 31 weather surveillance stations under the Central Weather Bureau. | NA; fit single-pollutant/ meteorological variable models. | *Percent change of hospital visits (95% CI) per 100-unit or 10-unit increase in 7-days lag mean exposures (overall results):* ▪PM_2.5_: 2.39% (1.11%, 3.67%) per 10 µg/m^3^ ↑  ▪NO: 15.97% (12.87%, 19.07%) per 10 ppb ↑  ▪NO_2_: 6.60% (4.51%, 8.69%) per 10 ppb ↑  ▪CO: 2.89% (1.47%, 4.31%) per 100 ppb ↑  ▪CH_4_: 4.76% (2.94%, 6.58%) per 100 ppb ↑ ▪NMHC: 4.38% (2.64%, 6.12%) per 100 ppb ↑ ▪Tmean: 6.37% (4.40%, 8.34%) per 10°C ↑ | ▪Also observed positive associations with rainfall, atmospheric pressure, and Ultraviolet B. |
| Choi, 2023 [50] | Cheongju and Sihwa-Banwol, South Korea, 2008-2019. | Retrospective cohort; Cox proportional hazards regression. | 1464 epilepsy cases, per ICD-10 classification (code G40), identified from the Korean National Health Insurance Service (NHIS) database. | Residential proximity to industrial complexes with high levels of PM_2.5_, PM_10_, NO_2_, SO_2_, O_3_, CO, heavy metals, VOCs, and PAHs. | Residence near polluting industrial complexes for > 5 years. | Age, sex, household income, and pre-existing comorbidities. | *HR (95% CI) for epilepsy among exposed vs control group (overall results):*  1.08 (1.00, 1.16) | ▪Focused exclusively on adults (aged > 40 years). ▪Stronger associations observed among older adults (aged >65 years), females, and those *without* preexisting diabetes. |
| Cui, 2017 [60] | Jinan, China, 2011-2013. | Daily time-series; Generalized Linear Model (GLM) with quasi-Poisson regression. | Medical emergency calls for convulsions and epilepsy at a medical emergency center. | Severe haze event in January 2013. | Daily, citywide mean concentrations from ground monitoring stations under the Chinese Ministry of Environmental Protection. | Daily PM_10_, Tmean, RH, year, day of week, and public holidays. | *RR (95% CI) for emergency calls for haze event compared with reference period:*  ▪ Convulsions: 1.50 (1.10, 1.90)  ▪ Epilepsy: 2.00 (1.30, 3.10) |  |
| Farahmandfard, 2022 [51] | Kerman, Iran, 2008-2020. | Ecological; Poisson Generalized Additive Model (GAM) with 7-day lags | 894 epilepsy admissions, per ICD-10 classification (code: G.40), identified from a single hospital registry | PM_2.5_, PM_10_, NO_2_, SO_2_, O_3_, and CO. | Daily, citywide mean estimates from ground monitoring stations of the Kerman Environmental Protection Agency. | Tmean and RH. | *Rate ratios (95% CI) for hospital admission per daily exposure (overall results):*  ▪Lag 0 O_3_: 0.98 (0.98, 0.99)  ▪Lag 0 NO_2_: 1.03 (1.02, 1.04)  ▪Lag 0 PM_2.5_: 1.00 (1.00, 1.01)  ▪Lag 0 PM_10_: 1.00 (1.00, 1.01)  ▪Lag 0 CO: 0.90 (0.79, 1.03) | ▪Stronger associations for NO_2_ and CO exposures on lag 0 among older adults (aged >59 years) and for CO on lag 4 among males. |
| Fluegge, 2021 [52] | USA, 2001-2005. | Annual time-series; Poisson regression. | Epilepsy cases per ICD-9 classification (codes 345, 780.3, 780.31, 780.32, 780.33, and 780.39) identified across 30 states under Healthcare Cost and Utilization Project. | PM_2.5_, PM_10_, NO_2_, N_2_O, SO_2_, and CO. | Annual, statewide mean measurement from ground monitoring stations maintained by the U.S. Environmental Protection Agency. | Adjusted for multiple pollutant comparisons. | *IRR (95% CI) of hospitalization per one log-unit increase in exposures (overall results):*  ▪N_2_O: 0.96 (0.93, 0.98)  ▪CO: 1.00 (0.98, 1.04)  ▪O_3_: 0.91 (0.83, 1.00)  ▪SO_2_: 0.99 (0.94, 1.03)  ▪NO_2_: 1.02 (0.97, 1.08)  ▪PM_10_: 0.97 (0.93, 1.02)  ▪PM_2.5_: 0.96 (0.87, 1.06) |  |
| Hjortebjerg, 2018 [30] | Denmark, 1995-2006. | Retrospective cohort; time-to-event analysis with Cox proportional hazards regression. | 2175 febrile seizure cases (from 51, 465 singleton births in a national birth cohort), per ICD-10 classification (code R56), identified from the nationwide Danish National Patient Register. | NO_2_ | Trimester-specific and annual, residence-level measurements from the Danish AirGIS dispersion modelling system. Noise pollution measured using SoundPLAN, a calculation program based on the Nordic prediction method. | Road traffic noise (for same exposure time-windows), sex of the child, maternal age at birth, parity, smoking or alcohol consumption during 1^st^ trimester, alcohol consumption during first trimester, level of education, disposable income one year before birth. | *IRR (95% CI) per IQR increase in exposure from fully adjusted models (overall results):*  ▪Mean NO_2_ exposure during pregnancy: 1.00 (0.96, 1.05) per 5.5 μg/m^3^ ↑ ▪Yearly NO_2_ residential exposure: 1.03 (0.99, 1.06) per 4 μg/m^3^ ↑ | ▪Focused exclusively on children (3months-5 years age) and on first diagnoses. ▪Excluded cases with preceding epilepsy.  ▪Accounted for residential mobility by assigning exposures at each residential address.  ▪ Observed interaction with parity. Stronger effects for uniparous (1 previous birth) and multiparous births (> 2 previous births) in a dose-response manner vs nulliparous birth (first child). |
| **Kawakami, 2020** [32] | Beppu, Japan, 2011-2018. | Weekly time-series; logistic regression. | 560 pediatric febrile seizure cases presenting at 1 medical center. | NO_2_, SO_2_, Tmean, atmospheric pressure, RH, rainfall amount, and sunshine duration. | Weekly, citywide mean estimates from the Oita Meteorological Monitoring Station of Japan Meteorological Agency. | Influenza virus infection, infectious gastroenteritis, and exanthem subitum (human herpesvirus 6 infection). | *OR (95% CI) per 10-unit increase in exposures (overall results):* ▪Tmean: 2.31 (0.99, 5.39) per 10°C ↑  ▪NO_2_: 0.35 (0.10, 1.13) per 10 ppb ↑  ▪SO_2_: 2.74 (0.16, 51.50) per 10 ppb ↑ | ▪Focused exclusively on children (6-60 months age) and on first diagnoses. ▪Excluded children with epilepsy, chromosomal abnormality, inborn errors of metabolism, hydrocephalus, brain tumor, intracranial hemorrhage, or history of intracranial surgery. ▪Adjusted for infectious risk factors. |
| **Kim, 2019** [34] | Changwon, South Korea, 2005-2018. | Daily time-series; distributed lag non-linear model (DLNM) with quasi-Poisson regression. | 1979 pediatric febrile seizure or febrile seizure plus cases, per ICD-10 classification (codes R56.0, G40.3), presenting at 1 medical center. | Tmean, average atmospheric pressure, and humidity according to lunar phase. | Daily, citywide measurement from the Korea Metrological Administration. | PM_10_, O_3_, NO_2_, CO, SO_2_, seasonality, long-term trends. | *Cumulative RR (95% CI) at specific exposure levels vs at median (overall results):* ▪No effect of Tmean ▪O_3_ at 1^st^ percentile (0.005 ppm): 0.51 (0.43, 0.61) ▪O_3_ at 25^th^ percentile (0.019 ppm): 0.77 (0.72, 0.83) ▪O_3_ at 50^th^ percentile (0.026 ppm): 0.94 (0.93, 0.96) ▪O_3_ at 75^th^ percentile (0.035 ppm): 1.23 (1.16, 1.29) ▪O_3_ at 99^th^ percentile (0.064 ppm): 2.84 (2.14, 3.77)  *Median of O_3_ and other exposures not reported | ▪Focused exclusively on children (<19 years age). ▪Generalized tonic-clonic seizure was most common presentation. |
| Lee, 2023 [53] | South Korea, 2016-2020. | Retrospective cohort; logistic regression. | 3190 pediatric epilepsy cases, per ICD-10 classification (codes: G40.0-G40.9), identified from the Korean National Health Insurance (NHI) claims database. | PM_2.5_, NO_2_, SO_2_, O_3_, CO, and heavy metals (Pb, Cd, Cr, Cu, Mn, Fe, Ni, and As). | Monthly average estimates from the Korea Environment Corporation assigned as maternal exposures across each trimester of pregnancy. | Maternal age, education level, infant sex, pregnancy period, household income, mutual co-pollutant adjustment. | *OR (95% CI) for epilepsy vs controls from five-pollutant model (overall results):*  ▪Pb during first trimester: 1.11 (1.04, 1.18) ▪Cd during third trimester: 2.193 (1.07, 4.48) |  |
| Min, 2023 [36] | New York, USA, 2010-2016. | Ecological; unconstrained distributed lag linear model (DLM) and Bayesian hierarchical model with a Poisson distribution. | 144,936 hospital admissions for convulsions, per Clinical Classification Software (CCS) scheme based on ICD-9 classification (CCS code: 83), identified from the New York Statewide Planning and Research Cooperative System (SPARCS). | PM_2.5_ | Annual, zip code-level estimates predicted at a resolution of 1 km × 1 km using an ensemble machine learning model using satellite data, meteorological variables, land-use variables, elevation, chemical transport model predictions, and several reanalysis data sets. | Urbanicity, population density as a measure of urbanicity, % female, median age, % White, % Black/African American, % uninsured adults, median household income, % children in poverty, % unemployed, educational attainment as % not graduated from high school among those 25 years of age and older, % smoker, % adults with obesity, and the number of primary care physicians per 100,000 people during the period 2010–2016. | *RR (95% Credible Interval) per 1 unit increase in exposure (overall results):*  ▪lag 0-1 years PM_2.5_: 1.04 (1.01, 1.06) per 1 μg/m^3^ ↑ | ▪Stronger subgroup effects in females and 'Other' races (American Indian/ Alaska Native, Asian, and Native Hawaiian/Pacific Islander). ▪Estimated excess hospital admission costs of convulsions due to exposures above the new WHO air quality guidelines (annual PM_2.5_ standard: 5 μg/m^3^): $153.73 (95% CI: 63.61, 244.19) million. |
| Radmanesh, 2019 [54] | Abadan, Iran, 2012-2016. | Daily time-series; Pearson Correlation. | 545 epilepsy patients, per ICD-10 classification, identified from Hospital Information Systems of 2 hospitals. | PM_10_ | Daily, citywide concentrations from 1 air quality monitoring station of the Abadan Environmental Protection Agency. | Wind speed and direction, air temperature, dew point, pressure, and RH. | *Pearson correlation between exposure and number of ED visits (at 1 hospital):* ▪Lag 0: 0.49 ▪Lag 1: 0.44 |  |
| Ronaldson, 2022 [55] | United Kingdom, 2010. | Cross-sectional assessment; Logistic regression. | Epilepsy cases self-reported and from record linkage with Hospital Episodes Statistics data (ICD-10 codes) in the UK Biobank cohort. | PM_2.5_, PM_10_, PMcoarse, and NO_2_ | Annual average estimates derived using a Land Use Regression (LUR) model developed as part of the European Study of Cohorts of Air Pollution Effects (ESCAPE). | Age, gender, ethnicity, education, employment status, household income, alcohol intake frequency, smoking status, physical activity, BMI, distance to nearest major road, traffic intensity on nearest major road, 24-h noise pollution, and residential greenspace. | *Adjusted OR (95% CI) for exposure quartile 4 vs quartile 1 from fully adjusted model (overall results):*  ▪NO_2_: 1.22 (0.98, 1.51) for exposure quartile 4 (>31.22 μg/m^3^) vs quartile 1 (<21.32 μg/m^3^) | ▪Focused on adults (aged 40-69 years at baseline). |
| Szyszkowicz, 2012 [39] | Vancouver, Canada, 1999-2003. | Time-stratified case-crossover; conditional logistic regression. | 2120 seizure cases presenting at a tertiary care hospital. | PM_2.5_, PM_10_, NO_2_, SO2, CO, and O_3._ | Daily, citywide concentrations from ground monitoring stations maintained by Environment Canada. | Tmean, RH, atmospheric pressure. | *OR (95% CI) per IQR increase in exposure:*  ▪Lag 1 PM_10_ (overall): 1.09 (1.01, 1.17) per 6.9 μg/m^3^ ↑ ▪Lag 4 PM_10_ (overall): 1.12 (1.04, 1.20) per 6.9 μg/m^3^ ↑ ▪Lag 1 PM_10_ (male): 1.09 (1.00, 1.18) per 6.9 μg/m^3^ ↑ ▪Lag 4 PM_10_ (male): 1.11 (1.01, 1.20) per 6.9 μg/m^3^ ↑ ▪Lag 1 SO_2_ (female): 1.15 (1.02, 1.28) per 1.9 ppb ↑ ▪Lag 2 SO_2_ (female): 1.18 (1.05, 1.32) per 1.9 ppb ↑ |  |
| Xu, 2016 [57] | Xi’an, China, 2013-2014. | Daily time series; Poisson regression. | 20,368 epilepsy outpatient-visits at a tertiary care hospital. | PM_10_, PM_2.5_, NO_2_, SO_2_, O_3_, and CO. | Daily, citywide mean concentrations from ground monitoring stations of China National Environmental Monitoring Center. | Long-term trends, day of the week, and daily Tmean and RH. | *Percent change (95% CI) in ED visits per 10 μg/m^3^ or 0.1 mg/m^3^ increase in lag day 0 exposures from single-pollutant models (overall results):*  ▪NO_2_: 3.17 (1.41, 4.93) per 10 μg/m^3^ ↑ ▪SO_2_: 3.55 (1.93, 5.18) per 10 μg/m^3^ ↑ ▪O_3_: -0.84 (-1.58, -0.09) per 10 μg/m3 ↑ ▪PM_10_: 0.14 (-0.13, 0.41) per 10 μg/m^3^ ↑ ▪PM_2.5_: 0.20 (-0.23, 0.62) per 10 μg/m^3^ ↑ ▪CO: 0.11 (-0.37, 0.59) per 0.1 μg/m^3^ ↑ | ▪NO_2_ and SO_2_ effects were stronger among children (aged <18 years). O_3_ effects were stronger among adults (aged 18-59 years). ▪Stronger SO_2_ and O_3_ effects in males, and NO_2_ in females.  ▪ Sensitivity-tested two-pollutant models.  NO_2_ and SO_2_ effects were stronger and retained significance after adjustment for  PM_2.5_. O_3_ effects became weaker and lost significance when adjusted for NO_2_. |
| **Yalçın, 2022** [58] | Diyarbakır, Turkey, 2009-2019. | Daily time-series; Poisson regression. | 1805 pediatric patients with epilepsy, per ICD-10 classification (codes: G40, G40.1, G40.2, G40.3, G40.4, G40.5, G40.6, G40.7, and 40.8, G40.9, G40.10, or Z82.0), presenting at a tertiary care hospital. | PM_10_, SO_2_, Tmean, RH, wind speed, total precipitation, and current pressure. | Daily, citywide mean measurements from the ground monitoring stations of the National Air Quality Monitoring Network and the Agriculture and Forestry General Directorate of Meteorology. | Seasonality, long-term trend, day of week, and public holiday. | *IRR (95% CI) per 10 unit or 1 unit increase in exposure (overall results):*  ▪PM_10_: 1.02 (1.02, 1.02) per 10 μg/m^3^ ↑ ▪SO_2_: 1.16 (1.15, 1.17) per 10 μg/m^3^ ↑ ▪Tmean: 1.03 (1.01, 1.06) per 1°C ↑ | Focused exclusively on children (aged <18 years). |
| **Yamaguchi, 2021** [42] | Kobe, Japan, 2011-2015. | Daily time-series; Poisson regression. | 97 pediatric patients of unprovoked seizures, admitted at a tertiary care hospital. | PM_10_, NO_2_, NO, SO_2_, OX, CH_4_, Tmean, atmospheric pressure, precipitation, RH, wind speed, and hours of sunlight. | Daily, citywide mean concentrations from ground monitoring stations of Hyogo Nanbu Taiki Sokutei Kyoku and the Japan Meteorology Agency. | Mutual co-exposure adjustment. | *Estimate (SE; p-value) per unit increase in exposures:*  ▪NO_2_: -42.2 (20.2; *p*-value = 0.04) per ppm ↑ ▪CH_4_: 7.20 (3.40; *p*-value = 0.03) per ppmC ↑  ▪SO_2_: 66.4 (91.9; *p*-value = 0.47) per ppm ↑  ▪NO: 33.2 (19.4; *p*-value = 0.09) per ppm ↑  ▪OX: 5.50 (13.8; *p*-value = 0.69) per ppm ↑  ▪Tmean: 0.01 (0.02; *p*-value = 0.59) per °C ↑ | Focused exclusively on children (aged <16 years). |
| **Yang, 2023** [43] | Hangzhou, China, 2018-2020. | Daily time-series; Logistic regression. | 775 children with convulsions, admitted at a tertiary care hospital. | AQI, PM_10_, PM_2.5_, NO_2_, SO_2_, CO, O_3_, Tmean, Tmin, Tmax, air humidity, wind force, precipitation. | Daily, citywide mean concentrations from ground monitoring stations of Hangzhou Environmental Inspection Center and Zhejiang Meteorological Bureau. | Mutual co-exposure adjustment. | *OR (95% CI) per unit increase in exposure (overall results):*  ▪SO_2_: 1.18 (1.10, 1.28) per μg/m^3^ ↑  ▪PM_10_: 0.99 (0.97, 1.00) per μg/m^3^ ↑ ▪PM_2.5_: 1.02 (1.00, 1.04) per μg/m^3^ ↑  ▪NO_2_: 1.00 (0.99, 1.01) per μg/m^3^ ↑  ▪CO: 1.60 (0.57, 4.49) per μg/m^3^ ↑  ▪O_3_: 1.00 (0.99, 1.00) per μg/m^3^ ↑  ▪Tmean: 0.85 (0.67, 1.08) per °C ↑  ▪Tmin: 1.05 (0.92, 1.21) per °C ↑  ▪Tmax: 1.12 (1.00, 1.30) per °C ↑ | ▪Focused exclusively on children (aged <5 years). ▪Monthly O_3_ exposures were negatively correlated (Pearson correlation = -0.44, *p*-value = 0.007) with monthly hospital visits for convulsions. |
| Zhou, 2023 [59] | Chongqing, China, 2014-2019. | Daily time series; quasi-Poisson generalized additive model. | 99,740 epilepsy outpatient visits, per ICD-10 classification (codes: G40 and G41), presenting at two tertiary care hospitals. | O_3_ | Daily, city-level mean estimates from 28 ground monitoring stations under the Chongqing Environmental Monitoring Center. | Day of week, holidays, PM_10_, PM_2.5_, SO_2_, CO, NO_2_, temperature, RH, sunlight hours, and rainfall. | *Percent change (95% CI) in ED visits per 10 μg/m^3^ increase in exposure from single-pollutant models (overall results):*  ▪Lag 0 O_3_: -0.519 (-0.954, -0.085) per 10 μg/m^3^ ↑ ▪Lag 1 O_3_: -0.567 (-1.002, -0.133) per 10 μg/m^3^ ↑ ▪Lag 2 O_3_: -0.150 (-0.578, 0.278) per 10 μg/m^3^ ↑ ▪Lag 3 O_3_: -0.241 (-0.676, 0.194) per 10 μg/m^3^ ↑ ▪Lag 0-1 average O_3_: -0.766 (-1.285, -0.247) per 10 μg/m^3^ ↑ ▪Lag 0-2 average O_3_: -0.697 (-1.283, -0.112) per 0.1 μg/m^3^ ↑ ▪Lag 0-3 average O_3_: -0.770 (-1.419, -0.122) per 0.1 μg/m^3^ ↑ | ▪Stronger subgroup effects among females, children (aged <18 years) and older adults (aged >65 years), and in the cool season.  ▪Sensitivity-tested two-pollutant models. In all age groups, the inverse association  remained significant at lag 0 and lag 1, and lag 01 to lag 03 after adjustment for other air pollutants. |

**Note:** Studies with first author and year in bold examined effects of both air pollution and temperature exposures.

**Table S3: Summary of studies examining relationships between temperature exposure and seizures and epilepsy.**

| **First author and publication year** | **Study location and duration** | **Study design and analytical approach** | **Study population and outcome(s)** | **Exposure(s)** | **Exposure resolution and assessment method** | **Covariate(s)** | **Main findings** | **Other findings/comment(s)** |
| --- | --- | --- | --- | --- | --- | --- | --- | --- |
| Bell, 2010 [46] | England and Wales, 1999-2000. | Retrospective cohort; compared observed vs expected deaths using chi-square goodness of fit test. | 409 SUDEP cases identified from the National Sentinel Clinical Audit of Epilepsy-Related Death. | Tmean and Tmin | Daily, townwide measurements (10 percentiles) from one weather station in Maidenhead town, southern England. | NA | *Observed vs expected number of deaths:* ▪Tmean (9th 10 percentile- second coldest): 53 vs 39.11  ▪Tmean (10th 10 percentile- coldest): 56 vs 42.46.  *Tmean range not reported. | ▪Excluded older adults (>60 years age). ▪Observed no associations with overall Tmean or Tmin. |
| Brás, 2018 [27] | Lisbon, Portugal, 2015. | Daily time-series; compared exposures on days without seizures with days with two or more seizures using Mann–Whitney test. | 307 seizure events (from 286 patients) presented at a tertiary care hospital. | Tmean, Tmin, Tmax, atmospheric pressure, RH, mean and maximum wind speed, and total precipitation. | Daily, citywide measurements from the Portuguese Institute for Sea and Atmosphere. | NA | *Exposure (median) on day without seizure vs days with >= 2 seizures:* ▪Tmean: 17.5°C vs 15°C (*p* value = 0.002) ▪Tmin: 14.2°C vs 11.3°C (*p* value = 0.001) ▪Tmax: 22.4°C vs 18.6°C (*p* value = 0.002) | ▪Focused on adults ▪Positive associations also observed with ↑ atmospheric pressure, ↑ maximum RH, winter days, and days with lower daylight duration. ▪Performed subgroup analyses for patients with first unprovoked seizure (observed no differences) and patients with previous seizures (observed similar results except for humidity). |
| Chang, 2019 [48] | Taiwan, (2000-NA). | Monthly time-series; Poisson regression. | 21,546 epilepsy cases, per ICD-9 classification (code 345) identified from the Bureau of National Health Insurance (NHI) claim database. | Tmean, accumulated precipitation, atmospheric pressure, RH, and number of hours of sunshine. | Monthly, citywide mean measurements from 25 weather surveillance stations under the Central Weather Bureau. | Co-exposure adjustment in multivariable model. | *RR (95% CI) of hospitalization per unit decrease in exposure (multivariable model):*  ▪Tmean: 1.02 (1.01, 1.02) per 1°C ↓ | ▪Focused on adults (>20 years age). ▪Identified threshold temperature for seizure prediction (Tmean <18 °C). |
| **Chiang, 2021** [29] | Taiwan, 2009-2013. | Daily time-series; Poisson regression. | 1,010,027 epileptic seizure events (from 180,175 epilepsy cases), per ICD-9 classification (codes 345 and 780.39), identified from the Bureau of National Health Insurance (NHI) claim database. | PM_2.5_, PM_10_, NO, NO_2_, SO_2_, O_3_, CO, CH_4_, NMHC, Tmean, maximum temperature difference, rainfall, RH, atmospheric pressure, wind speed, Ultraviolet B, pH scale of rain, electric conductivity of rain. | Daily, citywide mean concentrations from 77 air quality ground monitoring stations from the Taiwan Air Quality Monitoring Database and 31 weather surveillance stations under the Central Weather Bureau. | NA; fit single-pollutant/meteorological variable models. | *Percent change of hospital visits (95% CI) per 100-unit or 10-unit increase in 7-days lag mean exposures (overall results):* ▪PM_2.5_: 2.39% (1.11%, 3.67%) per 10 µg/m^3^ ↑  ▪NO: 15.97% (12.87%, 19.07%) per 10 ppb ↑  ▪NO_2_: 6.60% (4.51%, 8.69%) per 10 ppb ↑  ▪CO: 2.89% (1.47%, 4.31%) per 100 ppb ↑  ▪CH_4_: 4.76% (2.94%, 6.58%) per 100 ppb ↑ ▪NMHC: 4.38% (2.64%, 6.12%) per 100 ppb ↑ ▪Tmean: 6.37% (4.40%, 8.34%) per 10°C ↑ | ▪Also observed positive associations with rainfall, atmospheric pressure, and Ultraviolet B. |
| Izadyar, 2021 [31] | New York, USA, 2014-2017. | Daily time-series; Compared observed vs expected cases using chi-square goodness of fit test. | 119 inpatients with psychogenic nonepileptic seizures (PNES) admitted at 1 epilepsy monitoring unit. | Tmean and mean atmospheric pressure. | Daily, citywide measurements from the National Oceanic and Atmospheric Administration (NOAA). | NA | Observed no statistically significant cluster of seizures in any temperature category. | ▪Focused on adults (>18 years age).  ▪ Also observed positive association with daily mean atmospheric pressure. |
| **Kawakami, 2020** [32] | Beppu, Japan, 2011-2018. | Weekly time-series; logistic regression. | 560 pediatric febrile seizure cases presenting at 1 medical center. | NO_2_, SO_2_, Tmean, atmospheric pressure, RH, rainfall amount, and sunshine duration. | Weekly, citywide mean estimates from the Oita Meteorological Monitoring Station of Japan Meteorological Agency. | Influenza virus infection, infectious gastroenteritis, and exanthem subitum (human herpesvirus 6 infection). | *OR (95% CI) per 10-unit increase in exposures (overall results):* ▪Tmean: 2.31 (0.99, 5.39) per 10°C ↑  ▪NO_2_: 0.35 (0.10, 1.13) per 10 ppb ↑  ▪SO_2_: 2.74 (0.16, 51.50) per 10 ppb ↑ | ▪Focused exclusively on children (6-60 months age) and on first diagnoses. ▪Excluded children with epilepsy, chromosomal abnormality, inborn errors of metabolism, hydrocephalus, brain tumor, intracranial hemorrhage, or history of intracranial surgery. ▪Adjusted for infectious risk factors. |
| Kim, 2017 [33] | Changwon, South Korea, 2005-2015. | Daily time-series; distributed lag non-linear model (DLNM) with quasi-Poisson regression. | 108,628 ED visits for pediatric seizures, per ICD-10 classification (codes G40-G41, R56), presenting at 1 medical Center. | Tmean, Tmax, Tmin, DTR, humidity, atmospheric pressure, cloud cover, wind speed, and sunshine. | Daily, citywide measurements from the Korea Metrological Administration. | PM_10_, O_3_, NO_2_, CO, SO_2_, seasonality, long term trends. | *Cumulative RR (95% CI) at extreme weather points (vs median of Tmean: 15.9 °C) (overall results):*  ▪Tmean at 0.1th percentile (-4.8°C): 1.66 (1.04, 2.66) ▪Tmean at 5th percentile (0.4 °C): 1.46 (1.03, 2.08) ▪Tmean at 95th percentile (27.4 °C): 0.75 (0.58, 0.98) ▪Tmean at 99.9th percentile (30.1 °C): 0.71 (0.51, 0.97) | ▪Focused exclusively on children (<19 years age). ▪Assessed subgroup effects by seizure type: febrile seizure, afebrile seizures, seizure of known epilepsy, and status epilepticus. ▪Only febrile seizure (inversely) affected by Tmean. |
| **Kim, 2019** [34] | Changwon, South Korea, 2005-2018. | Daily time-series; distributed lag non-linear model (DLNM) with quasi-Poisson regression. | 1979 pediatric febrile seizure or febrile seizure plus cases, per ICD-10 classification (codes R56.0, G40.3), presenting at 1 medical center. | Tmean, average atmospheric pressure, and humidity according to lunar phase. | Daily, citywide measurements from the Korea Metrological Administration. | PM_10_, O_3_, NO_2_, CO, SO_2_, seasonality, long-term trends. | *Cumulative RR (95% CI) at specific exposure levels vs at median (overall results):* ▪No effect of Tmean ▪O_3_ at 1st percentile (0.005 ppm): 0.51 (0.43, 0.61) ▪O_3_ at 25th percentile (0.019 ppm): 0.77 (0.72, 0.83) ▪O_3_ at 50th percentile (0.026 ppm): 0.94 (0.93, 0.96) ▪O_3_ at 75th percentile (0.035 ppm): 1.23 (1.16, 1.29) ▪O_3_ at 99th percentile (0.064 ppm): 2.84 (2.14, 3.77)  *Median of O_3_ and other exposures not reported. | ▪Focused exclusively on children (<19 years age). ▪Generalized tonic-clonic seizure was most common presentation. |
| Lin, 2018 [35] | Taiwan, 2007-2013. | Daily time-series data mining; ensemble empirical mode decomposition (EEMD) method and Fourier–Gaussian decomposition (FGD) algorithm. | 80,554 records of epileptic seizures identified from the emergency medical service system managed by the Ministry of Health and Welfare. | Accumulated precipitation, Tmax, Tmin, RH, maximum RH, mean wind speed/direction, station pressure, maximum station pressure, minimum station pressure, and sunshine duration. | Daily, citywide measurements from the Central Weather Bureau. | Minimum RH. | ▪Negative Pearson correlations with Tmax across multiple cities; ranging from −0.38 in Kaohsiung to −0.26 in Taichung  ▪Negative Pearson correlations with Tmin across multiple cities; ranging from −0.35 in Nantou to −0.23 in Chiayi. | ▪Also found positive correlations with atmospheric pressure across multiple cities, ranging from 0.218 in Keelung to 0.328 in Nantou. |
| Rakers, 2017 [37] | Jena, Germany, 2003-2010. | Bidirectional case-crossover; conditional logistic regression. | 604 inpatients with epileptic seizures, per ICD-10 classification (codes G40.x), admitted at a tertiary care hospital. | Tmean, RH, and atmospheric pressure. | Daily, citywide measurements from 1 weather station of the of the Jena University of Applied Sciences. | NA | *OR (95% CI) (overall results):* ▪Lag 1 Tmean >20°C: 0.54 (0.32, 0.90) | ▪Focused on adults (>18 years age). |
| Rüegg, 2008 [38] | Basel, Switzerland, 2003-2006 | Daily time-series; Poisson regression and likelihood ratio tests. | 184 patients diagnosed with status epilepticus admitted to a tertiary care center ICU. | Tmean, RH, barometric pressure, and wind force. | Daily, citywide measurements from weather stations of Meteo Switzerland. | Weekly cycles and influences of the other meteorological variables. | *IRR (95% CI) per unit increase in exposure (overall results):*  ▪Tmean: 0.98 (0.96, 1.00) per 1°C ↑ |  |
| Sun, 2022 [56] | Hefei, China, 2015-2020 | Daily time-series; quasi-Poisson regression combined with distributed lag non-linear model (DLNM). | 28,020 epilepsy-related clinic visits, per ICD-10 classification (codes: G40-41, R56) at a tertiary care hospital. | Apparent Temperature. | Daily, citywide mean measurements from the China National Meteorological Information Center. | Day of the week, holidays, seasonal and long-term trends, RH, and PM_2.5._ | *RR (95% CI) at the 5th percentile (-1.5°C) compared to 17°C (overall results):*  ▪Lag 1: 1.06 (1.02, 1.10) ▪Lag 2: 1.05 (1.02, 1.08) ▪Lag 3: 1.05 (1.02, 1.07) ▪Lag 4: 1.04 (1.02, 1.07) ▪Lag 5: 1.04 (1.01, 1.07) ▪Lag 6: 1.03 (1.00, 1.06) | ▪Stronger effects of lower AT in younger age groups (0-14 years and 15-29 years). |
| Treib, 2021 [40] | Kaiserslautern, Germany, 2015-2019. | Daily time-series; conditional Poisson regression. | 2,813 epileptic seizure cases presenting at a tertiary care hospital. | Tmax, Tmean, Tmin, peak, RH, precipitation mean vapor pressure, mean barometric pressure, duration of sunshine, mean wind intensity, mean complete cloud cover, and maximum wind speed. | Daily, citywide measurements from two weather stations maintained by the National Meteorological Service. | Year, month, and day of the week. | *IRR (95% CI) per unit increase in exposure (overall results):*  ▪Tmax: 1.00 (0.99, 1.01) per 1°C ↑ ▪Tmean: 1.00 (0.99, 1.02) per 1°C ↑ ▪Tmin: 1.01 (1.00, 1.02) per 1°C ↑ |  |
| Woo, 2018 [41] | Seoul, South Korea, 2009-2013. | Daily time-series; Poisson generalized additive model. | 29,240 children diagnosed with febrile seizures, per ICD-10 classification (codes: R56.0, G41, R56.8), at a tertiary care hospital, identified through record-linkage with the Korea National Health Insurance Review and Assessment Service. | Tmean, mean humidity, mean sea-level pressure, and mean amount of precipitation. | Daily, citywide measurements from the Korea Meteorological Administration. | NA | Seizure risk increased bimodally with Tmean; between -7°C to -1°C and 18°C to 21°C (effect estimates not reported). | ▪Focused exclusively on children (<5 years age) and on first diagnoses. ▪Also, seizure risk increased when sea-level pressure was low at 997–1,010 hPa.  ▪Also, seizure risk was high when humidity was 20–45% and was low when humidity was as high as 90%. |
| **Yalçın, 2022** [58] | Diyarbakır, Turkey, 2009-2019. | Daily time-series; Poisson regression. | 1805 pediatric patients with epilepsy, per ICD-10 classification (codes: G40, G40.1, G40.2, G40.3, G40.4, G40.5, G40.6, G40.7, and 40.8, G40.9, G40.10, or Z82.0), presenting at a tertiary care hospital. | PM_10_, SO_2_, Tmean, RH, wind speed, total precipitation, and current pressure. | Daily, citywide mean measurements from the ground monitoring stations of the National Air Quality Monitoring Network and the Agriculture and Forestry General Directorate of Meteorology. | Seasonality, long-term trend, day of week, and public holiday. | *IRR (95% CI) per 10 unit or 1 unit increase in exposure (overall results):*  ▪PM_10_: 1.02 (1.02, 1.02) per 10 μg/m^3^ ↑ ▪SO_2_: 1.16 (1.15, 1.17) per 10 μg/m^3^ ↑ ▪Tmean: 1.03 (1.01, 1.06) per 1°C ↑ | Focused exclusively on children (aged <18 years). |
| **Yamaguchi, 2021** [42] | Kobe, Japan, 2011-2015. | Daily time-series; Poisson regression. | 97 pediatric patients of unprovoked seizures, admitted at a tertiary care hospital. | PM_10_, NO_2_, NO, SO_2_, OX, CH_4_, Tmean, atmospheric pressure, precipitation, RH, wind speed, and hours of sunlight. | Daily, citywide mean concentrations from ground monitoring stations of Hyogo Nanbu Taiki Sokutei Kyoku and the Japan Meteorology Agency. | Mutual co-exposure adjustment. | *Estimate (SE; p-value) per unit increase in exposures:*  ▪NO_2_: -42.2 (20.2; *p*-value = 0.04) per ppm ↑ ▪CH_4_: 7.20 (3.40; *p*-value = 0.03) per ppmC ↑  ▪SO_2_: 66.4 (91.9; *p*-value = 0.47) per ppm ↑  ▪NO: 33.2 (19.4; *p*-value = 0.09) per ppm ↑  ▪OX: 5.50 (13.8; *p*-value = 0.69) per ppm ↑  ▪Tmean: 0.01 (0.02; *p*-value = 0.59) per °C ↑ | Focused exclusively on children (aged <16 years). |
| **Yang, 2023** [43] | Hangzhou, China, 2018-2020. | Daily time-series; logistic regression. | 775 children with convulsions, admitted at a tertiary care hospital. | AQI, PM_10_, PM_2.5_, NO_2_, SO_2_, CO, O_3_, Tmean, Tmin, Tmax, air humidity, wind force, precipitation. | Daily, citywide mean concentrations from ground monitoring stations of Hangzhou Environmental Inspection Center and Zhejiang Meteorological Bureau. | Mutual co-exposure adjustment. | *OR (95% CI) per unit increase in exposure (overall results):*  ▪SO_2_: 1.18 (1.10, 1.28) per μg/m^3^ ↑  ▪PM_10_: 0.99 (0.97, 1.00) per μg/m^3^ ↑ ▪PM_2.5_: 1.02 (1.00, 1.04) per μg/m^3^ ↑  ▪NO_2_: 1.00 (0.99, 1.01) per μg/m^3^ ↑  ▪CO: 1.60 (0.57, 4.49) per μg/m^3^ ↑  ▪O_3_: 1.00 (0.99, 1.00) per μg/m^3^ ↑  ▪Tmean: 0.85 (0.67, 1.08) per °C ↑  ▪Tmin: 1.05 (0.92, 1.21) per °C ↑  ▪Tmax: 1.12 (1.00, 1.30) per °C ↑ | ▪Focused exclusively on children (aged <5 years). ▪Monthly O_3_ exposures were negatively correlated (Pearson correlation = -0.44, *p*-value = 0.007) with monthly hospital visits for convulsions. |
| Zhang, 2023 [44] | Brazil, 2000-2015 | Time-stratified case-crossover; distributed lag non-linear model with conditional logistic regression. | 225,699 hospitalizations for epileptic seizures across 1816 municipalities in 24 states, per ICD-10 classification (codes: G40.1-G40.9 and G41.1, G41.2, G41.8, and G41.9), from the Brazilian Unified Health System. | Tmean, | Daily, municipality-level estimates from a national dataset of 0.25° x 0.25° resolution, based on measurements from 735 weather stations with the inverse distance weighting approach. | RH and holidays. | *OR (95% CI) per unit increase in exposure (overall results):*  ▪Tmean (cumulative 0-7 lag days): 1.04 (1.03, 1.06) per 1°C ↑ above the heat impact threshold of 26°C | ▪Focused exclusively on hot season exposures. ▪Stronger subgroup effects in females, individuals aged 20-39 years, and persons living in *high-income* regions. |

**Note:** Studies with first author and year in bold examined effects of both air pollution and temperature exposures.
